# Uncovering miRNA–mRNA Regulatory Networks Related to Olaparib Resistance and Resensitization of *BRCA2*^MUT^ Ovarian Cancer PEO1-OR Cells with the ATR/CHK1 Pathway Inhibitors

**DOI:** 10.3390/cells13100867

**Published:** 2024-05-17

**Authors:** Łukasz Biegała, Damian Kołat, Arkadiusz Gajek, Elżbieta Płuciennik, Agnieszka Marczak, Agnieszka Śliwińska, Michał Mikula, Aneta Rogalska

**Affiliations:** 1 Department of Medical Biophysics, Institute of Biophysics, Faculty of Biology and Environmental Protection, University of Lodz, Pomorska 141/143, 90-236 Lodz, Poland; lukasz.biegala@edu.uni.lodz.pl (Ł.B.); arkadiusz.gajek@biol.uni.lodz.pl (A.G.); agnieszka.marczak@biol.uni.lodz.pl (A.M.); 2 Doctoral School of Exact and Natural Sciences, University of Lodz, Jana Matejki 21/23, 90-237 Lodz, Poland; 3 Department of Functional Genomics, Medical University of Lodz, Żeligowskiego 7/9, 90-752 Lodz, Poland; damian.kolat@umed.lodz.pl (D.K.); elzbieta.pluciennik@umed.lodz.pl (E.P.); 4 Department of Biomedicine and Experimental Surgery, Medical University of Lodz, Narutowicza 60, 90-136 Lodz, Poland; 5 Department of Nucleic Acid Biochemistry, Medical University of Lodz, Pomorska 251, 92-213 Lodz, Poland; 6 Department of Genetics, Maria Sklodowska-Curie National Research Institute of Oncology, Roentgena 5, 02-781 Warsaw, Poland; michal.mikula@nio.gov.pl

**Keywords:** ovarian cancer, miRNA profiling, olaparib, resistance, ATR/CHK1 pathway, combination therapy, growth factors, bioinformatics, TCGA data

## Abstract

Resistance to olaparib is the major obstacle in targeted therapy for ovarian cancer (OC) with poly(ADP-ribose) polymerase inhibitors (PARPis), prompting studies on novel combination therapies to enhance olaparib efficacy. Despite identifying various mechanisms, understanding how OC cells acquire PARPi resistance remains incomplete. This study investigated microRNA (miRNA) expression in olaparib-sensitive (PEO1, PEO4) and previously established olaparib-resistant OC cell lines (PEO1-OR) using high-throughput RT-qPCR and bioinformatic analyses. The role of miRNAs was explored regarding acquired resistance and resensitization with the ATR/CHK1 pathway inhibitors. Differentially expressed miRNAs were used to construct miRNA–mRNA regulatory networks and perform functional enrichment analyses for target genes with miRNet 2.0. TCGA-OV dataset was analyzed to explore the prognostic value of selected miRNAs and target genes in clinical samples. We identified potential processes associated with olaparib resistance, including cell proliferation, migration, cell cycle, and growth factor signaling. Resensitized PEO1-OR cells were enriched in growth factor signaling via PDGF, EGFR, FGFR1, VEGFR2, and TGFβR, regulation of the cell cycle via the G2/M checkpoint, and caspase-mediated apoptosis. Antibody microarray analysis confirmed dysregulated growth factor expression. The addition of the ATR/CHK1 pathway inhibitors to olaparib downregulated FGF4, FGF6, NT-4, PLGF, and TGFβ1 exclusively in PEO1-OR cells. Survival and differential expression analyses for serous OC patients revealed prognostic miRNAs likely associated with olaparib resistance (miR-99b-5p, miR-424-3p, and miR-505-5p) and resensitization to olaparib (miR-324-5p and miR-424-3p). Essential miRNA–mRNA interactions were reconstructed based on prognostic miRNAs and target genes. In conclusion, our data highlight distinct miRNA profiles in olaparib-sensitive and olaparib-resistant cells, offering molecular insights into overcoming resistance with the ATR/CHK1 inhibitors in OC. Moreover, some miRNAs might serve as potential predictive signature molecules of resistance and therapeutic response.

## 1. Introduction

Olaparib, a widely-used oral poly(ADP-ribose) polymerase inhibitor (PARPi), has demonstrated efficacy in the therapy for ovarian cancer (OC), especially high-grade serous ovarian cancer (HGSOC) with germline *BRCA1/2* mutations [1]. HGSOC is a grade 3 subtype of epithelial OC accounting for 70–80% of deaths and is often diagnosed at advanced stages [2]. Despite olaparib’s efficacy in recurrent platinum-sensitive OC [3], acquired resistance to PARPi poses a significant challenge [4]. Indeed, there is a need for a precise characterization of olaparib sensitivity since some OC patients without *BRCA1/2* mutation or associated homologous recombination deficiency (HRD) may respond to PARPi. Therefore, an understanding of the molecular mechanisms underlying resistance and sensitivity to olaparib is urgently needed. Over recent years novel olaparib combinations have been evaluated to combat associated resistance and improve outcomes in OC patients. For instance, the addition of some agents, including antiangiogenic molecules or the ATR/CHK1 pathway inhibitors, has shown beneficial antitumor effects clinically in OC [5,6]. The ATR/CHK1 pathway is involved in multiple aspects of the DNA damage response, including the activation of cell cycle checkpoints, the stabilization of stalled replication forks, and the regulation of DNA repair pathways [7,8]. Given the role of the ATR and CHK1 kinases in protecting genomic integrity, targeted inhibition of the ATR/CHK1 axis constitutes a promising strategy to enhance olaparib efficacy in HGSOC. Recently, it was reported that olaparib combined with the ATR/CHK1 pathway inhibitors exerts synergistic antitumor activity in olaparib-resistant HGSOC cell lines with *BRCA1*/2 mutations [7,9]. In the most recent findings from the CAPRI trial, six partial responses were observed in homologous-recombination-deficient platinum-sensitive recurrent HGSOC when olaparib was combined with the ATRi ceralasertib. This response was seen among 12 patients who were eligible for efficacy evaluation and had progressed after prior PARPi treatment [10]. However, there is still a need to unravel molecular basics explaining acquired resistance to olaparib and mechanisms associated with the resensitization of OC cells to PARPi.

MicroRNAs (miRNAs) are short non-coding RNAs that act as major post-transcriptional regulators of gene expression. Mature miRNAs can serve as oncogenes or tumor suppressors based on their modulating effect on target genes [11]. Mechanistically, a single miRNA can concurrently regulate multiple genes, and each gene can be affected by many miRNAs forming complex regulatory networks involved in diverse biological processes [12]. Aberrant miRNA expression has been linked to processes such as genomic instability, tumor progression, metastasis, and chemosensitivity in OC [11]. Numerous studies demonstrated the role of specific circulating miRNAs as diagnostic biomarkers in OC screening [13]. A model established by Yokoi et al. showed extremely high sensitivity and specificity for the detection of OC based on the expression of ten serum miRNAs [14]. Recently, a growing number of studies have demonstrated regulatory networks linking miRNAs and genes associated with resistance to cisplatin in OC [15,16]. However, the miRNA–mRNA regulations in *BRCA2*^MUT^ olaparib-resistant HGSOC cells resensitized with the ATR/CHK1 pathway inhibitors have not been reported till now.

Our in vitro study unveils potential post-transcriptional mechanisms involved in acquiring resistance to olaparib in OC cells and their resensitization through combination treatments. In this work, differentially expressed (DE) miRNAs were linked to olaparib resistance and to response to olaparib combined with ATR/CHK1 pathway inhibitors in the PEO1-OR olaparib-resistant *BRCA2*^MUT^ HGSOC cell line in vitro using RT-qPCR profiling. The network-based approach revealed target genes and their potential biological roles through functional enrichment analyses. Importantly, dysregulation of growth factors and their receptor expression was confirmed in olaparib-resistant PEO1-OR cells. The clinical relevance of selected miRNAs and their target genes was studied in TCGA-OV dataset by Kaplan–Meier survival analysis, stage-wise differential expression analysis, co-expression analysis, and comparison of gene expression in normal ovarian tissue and OC tumors.

## 2. Materials and Methods

### 2.1. Materials

Olaparib (O) was purchased from Selleck Chemicals (Houston, TX, USA). ATRi (A, ceralasertib) and CHK1i (C, MK-8776) were purchased from Wuhan ChemNorm Biotech (Wuhan, China). The inhibitors were dissolved in 100% dimethyl sulfoxide (DMSO) to create stock solutions, which were then stored at −80 °C for a maximum of 6 months. Cell culture reagents were purchased from Thermo Fisher Scientific (Waltham, MA, USA). Chemicals and solvents were purchased from Merck Life Science (Poznań, Poland) or Avantor Performance Materials Poland (Gliwice, Poland). The remaining reagents utilized in the study are detailed in the following sections of the Materials and Methods (Section 2), as well as in Table S1.

### 2.2. Cell Lines and Treatment

The human HGSOC cell lines, PEO1 (*BRCA2*^MUT^) and PEO4 (*BRCA2*^REV^), which are sensitive to olaparib [7,17], were purchased from the ECACC (Salisbury, UK). Both cell lines were developed from the same patient before (PEO1) and after (PEO4) development of clinical platinum resistance representing disease progression [18,19]. Acquired resistance to olaparib in the PEO1-OR (*BRCA2*^MUT^) human HGSOC cell line, with a double mutation in *BRCA2*, was previously established through continuous exposure of PEO1 cells to gradually escalating doses of olaparib, as detailed in a prior report [17]. Cells were grown as monolayers in RPMI 1640 medium containing GlutaMAX™ supplement, HEPES, and 10% FBS (37 °C, 5% CO_2_) and were routinely subcultured using 0.1% trypsin solution with 0.4 mM EDTA.

Before treatment, cells were seeded in 100 mm dishes (2 × 10^6^ cells) and incubated for 24 h (37 °C, 5% CO_2_). The next day culture medium was changed, and cells were incubated with tested inhibitors or their combinations for 2 days (37 °C, 5% CO_2_) at concentrations previously established to exert synergistic antitumor activity [7]. PEO1 and PEO4 olaparib-sensitive cells were incubated with 10 μM olaparib, 5 μM ATRi, and 1 μM CHK1i, whereas PEO1-OR olaparib-resistant cells were incubated with 15 μM olaparib, 7.5 μM ATRi, and 2.5 μM CHK1i. Following the incubation, cells were harvested by trypsinization, centrifuged (300× *g*, 5 min, 4 °C), washed in ice-cold PBS, and stored as pellets at −80 °C before total protein extraction and RNA isolation. Cell culture experiments were independently repeated four times (*n* = 4).

### 2.3. RNA Isolation

Isolation of small-RNA-containing total RNA from four independent sets of samples (*n* = 4) was performed using a *mir*Vana™ miRNA Isolation Kit (Thermo Fisher Scientific, Waltham, MA, USA) according to the manufacturer’s instructions. At the end of the procedure, RNA was eluted from the glass-fiber filters using 50 μL of nuclease-free water pre-heated to 95 °C and stored in aliquots at −80 °C. The quality and quantity of isolated RNA were analyzed by absorbance measurements at 230, 260, and 280 nm using a BioTek Eon™ microplate spectrophotometer (BioTek Instruments, Winoosk, VT, USA).

### 2.4. RT-qPCR Global miRNA Expression Profiling with Predesigned TaqMan™ Array Human MicroRNA Cards in HGSOC Cell Lines

To preliminarily identify differentially expressed (DE) miRNAs, quantitative real-time PCR (qRT-PCR) global expression profiling of 754 unique human miRNAs was performed using pre-designed TaqMan™ Array Human MicroRNA A+B Cards Set v3.0 (Thermo Fisher Scientific, Waltham, MA, USA) according to the manufacturer’s instructions (Part Number 4399721 Revision C from 07/2010 and Part Number 4399813 Revision D from 11/2018). The screening analysis of miRNA profiles in PEO1 and PEO1-OR cell lines was performed using equivalent amounts of total RNA pooled from four biological replicates for each condition-specific sample to obtain one cDNA sample for each treatment condition.

Firstly, miRNAs were reverse transcribed using 1000 ng of total RNA with the TaqMan™ MicroRNA Reverse Transcription Kit and Megaplex™ RT Primers Human Pool Set v3.0 (Thermo Fisher Scientific, Waltham, MA, USA) in the final reaction volume of 7.5 μL in a PTC-200 DNA Engine^®^ Cycler (MJ Research Inc., St. Bruno, QC, Canada). Nuclease-free water was used instead of RNA to prepare no template control (NTC) reactions. The thermal-cycling conditions were as follows: 40 cycles of 16 °C for 2 min, 42 °C for 1 min, and 50 °C for 1 min, followed by 85 °C for 5 min and cooling at 4 °C. The ramping speed was set to 3 °C/s and the lid temperature was set to 105 °C. The obtained cDNA (133.3 ng/μL) was stored undiluted at −80 °C.

Sample-specific PCR reaction mixes were prepared to perform qRT-PCR reactions by mixing 450 µL of 2 × TaqMan™ Universal Master Mix II with no UNG, 444 µL of nuclease-free water, and 6 µL of the reverse transcription product (separate for Megaplex RT pools A and B) in the final reaction volume of 900 µL. Each of the eight TaqMan^®^ Low-density Array (TLDA) ports for both A and B cards was filled with 100 µL of a PCR reaction mix. The qPCR reactions were run in a 7900HT Fast Real-Time PCR System (Thermo Fisher Scientific, Waltham, MA, USA) using the default thermal-cycling conditions (94.5 °C for 10 min followed by 40 cycles of 97 °C for 30 s and 59.7 °C for 1 min). Raw cycle-threshold (C_T_) values were collected using automatic baseline settings and a threshold of 0.2. Informative target miRNAs were defined based on expression in untreated cells as having C_T_ value < 35.

Relative qPCR analysis was performed in DataAssist™ v3.01 software. The relative expression of 754 miRNAs was calculated using the comparative 2^−∆∆Ct^ method [20] and global mean normalization (median C_T_ values of all miRNAs with C_T_ < 35 in each sample as the normalization factor) recommended for large-scale expression profiling [21,22]. Outliers within technical replicates were excluded from data analysis calculations by the software. Relative miRNA levels are expressed as fold changes (FCs) relative to untreated control cells. Preliminary selection of the most relevantly dysregulated miRNAs for further validation and studies assumed that miRNA was differentially expressed (up- or downregulated) with an absolute FC of at least 1.5. This FC cut-off enables the detection of subtle changes that cumulatively might have an impact on cell biology [23,24,25].

Next, bioinformatics selection of dysregulated miRNAs was performed to select key miRNAs for validation on the Custom TaqMan^®^ Array MicroRNA Cards fitting 44 target miRNAs and 3 endogenous controls. Briefly, all dysregulated miRNAs were used for network-based analyses with the web tool MIENTURNET (http://userver.bio.uniroma1.it/apps/mienturnet/; accessed on 11 January 2023) [26]. The network was filtered with the default settings (thresholds for the minimum number of miRNA–target interactions of two and false-discovery rate of one). Dysregulated miRNAs were prioritized based on strong experimental evidence of miRNA–target interactions from miRTarBase (Release 7.0, September 2017) [27] and calculated miRNA node degree in the network.

### 2.5. RT-qPCR Validation of Dysregulated miRNA Expression with Custom TaqMan™ Array MicroRNA Cards in HGSOC Cell Lines

The expression of 44 selected miRNAs was validated using Custom TaqMan™ Array MicroRNA Cards (Thermo Fisher Scientific, Waltham, MA, USA) according to the manufacturer’s instructions (Publication Part no. 4478705 Revision A from 01/2013). Customized cards were designed for 44 miRNAs of interest and 3 candidate endogenous control assays (U6 snRNA in duplicate, RNU48 snoRNA, and miR-30e-3p). The stability of endogenous control genes’ expression was confirmed in all OC cell lines, and all three were used as normalizers since it is a preferred approach for miRNA normalization [24,25]. Details are included in the Supplementary Methods. The expression levels of selected miRNA were calculated in all individual samples (control, O, A, C, O + A, and O + C) and all HGSOC cell lines (PEO1, PEO1-OR, and PEO4) using total RNA from 4 independent biological replicates (*n* = 4).

Firstly, miRNAs were reverse transcribed for each sample separately (biological replicate) using 1000 ng of total RNA with the TaqMan™ MicroRNA Reverse Transcription Kit and Custom RT Primer Pool composed of individual RT primers for each target provided with the Custom TaqMan™ Array MicroRNA Cards (Thermo Fisher Scientific, Waltham, MA, USA). RT reactions were run in the final reaction volume of 15 μL according to the manufacturer’s instructions in a PTC-200 DNA Engine^®^ Cycler (MJ Research Inc., St. Bruno, QC, Canada). NTC samples used nuclease-free water in place of RNA in the RT reaction. The thermal-cycling conditions were as follows: 16 °C for 30 min, 30 °C for 30 min, 85 °C for 5 min, followed by cooling at 4 °C. The ramping speed was set to 2.5 °C/s and the lid temperature was set to 105 °C to prevent condensation. The obtained cDNA (66.7 ng/μL) was stored undiluted at −80 °C.

Following the RT step, 3 µL of the RT reaction product (200 ng cDNA) was combined with 52 µL of nuclease-free water and 55 µL of 2 × TaqMan™ Universal Master Mix II with no UNG. TLDA ports were filled with 100 µL of sample-specific PCR reaction mixes. Untreated control samples were run in duplicate, whereas treated samples and NTC samples were run in one technical replicate on one card. The qPCR reactions were run in a 7900HT Fast Real-Time PCR System (Thermo Fisher Scientific, Waltham, MA, USA) using the default thermal-cycling conditions (94.5 °C for 10 min followed by 40 cycles of 97 °C for 30 s and 59.7 °C for 1 min). Raw C_T_ values were collected using automatic baseline settings and a threshold of 0.2 and exported using DataAssist™ v3.01 software. Informative target miRNAs were defined based on expression in untreated control samples as having C_T_ value < 32 in ≥75% of samples (at least 3 out of 4 biological replicates). C_T_ values ≥ 35 for miRNAs in treated samples were included in calculations if miRNA was defined as detected in untreated control cells as these values contain important biological information. Individual C_T_ values were averaged for control samples run in duplicate.

Relative miRNA expression was calculated as FC compared to untreated cells using the comparative 2^−∆∆Ct^ method. Log-transformed relative quantity data (log_2_ of FC) were used for statistical analysis. Expression of all miRNAs was visualized with a heatmap generated with GraphPad Prism. Clustering of samples with similar informative miRNA expression was performed with the ClustVis web tool using correlation distance and average linkage [28]. Significantly dysregulated miRNAs were defined as differentially expressed (up- or downregulated) with an absolute FC ≥ 1.5 (log_2_ of FC ≥ 0.585) and *p* < 0.05.

### 2.6. Construction and Analysis of miRNA–mRNA Regulatory Networks

A regulatory network between significantly dysregulated miRNAs and their target mRNAs was constructed and analyzed using the miRNet 2.0 web-based platform [29] (accessed on 7 December 2023) and two miRNA databases with experimentally validated interactions (miRTarBase v8.0 and TarBase v8.0). The miRNA–mRNA network was integrated with protein–protein interaction (PPI) network of genes to provide deeper insight into regulatory mechanisms. The original network was simplified by reducing less important nodes and edges using a minimum network algorithm to focus on key connectivity according to the recommendations for the exploration of complex networks [29].

### 2.7. Functional Enrichment Analysis

To interpret the interactions and predict functional pathways for target genes, enrichment analyses were performed with a hypergeometric test algorithm using the Reactome database and Gene Ontology (GO) terms of the biological processes (GO:BP). Adjusted *p* value < 0.05 was set as the cut-off to select fifteen significantly enriched terms and pathways with the most hits. Cytoscape software (version 3.10.1) was employed to further customize, visualize, and analyze regulatory networks. The cytoHubba plug-in was used to identify hub nodes from the miRNA–mRNA network based on the maximal clique centrality (MCC) algorithm which ranks genes within the network. Out of all nodes, the top 10 genes with the highest connectivity were assigned as potential hub genes. Subnetworks of the miRNA–hub genes were constructed to visualize core interactions.

### 2.8. Growth Factor Expression Profiling with Antibody Array

The expression of 41 human growth factors (GFs) and their receptors was semi-quantitatively determined in PEO1 and PEO1-OR cells incubated with tested inhibitors or their combinations for 48 h using commercially available RayBio^®^ C-Series Human Growth Factor Antibody Array 1 (RayBiotech Life, Inc., Peachtree Corners, GA, USA) as described previously [7]. Each array was loaded with a sample containing 150 μg of total protein and processed in accordance with the manufacturer’s instructions.

The changes in protein expression were calculated as a fold change compared to untreated control cells. Each experiment was conducted independently twice, with two technical replicates on each membrane, and the results were presented as mean ± SD (*n* = 4).

### 2.9. Differential Expression Analysis in Ovarian Cancer Patients

Stage-wise differential expression analysis was performed using The Cancer Genome Atlas (TCGA) repository to search and download a filtered dataset of OC patients with available clinical data as well as mRNA and miRNA expression quantification data generated using the STAR workflow [30]. The analysis was performed for serous OC samples from patients with stages II (*n* = 14), III (*n* = 125), and IV (*n* = 17) who underwent only pharmaceutical therapy since radiation therapy is currently rarely used [31,32]. Read counts were pre-processed via calcNormFactors and subjected to differential expression analysis using the limma-voom method [33]. Genes and miRNAs with counts per million (CPM) < 10 in ≥50% of samples were filtered out as the minimal cut-off for biological relevance. Outliers were identified according to the ROUT method with 1% FDR using GraphPad Prism. The results were visualized by medians with box-and-whisker plots extending from the 25th to 75th percentiles. Statistical significance was evaluated with one-way ANOVA followed by the Tukey multiple comparison test (normally distributed data), or Kruskal–Wallis test followed by Dunn’s multiple comparison test (non-normally distributed data). Pairwise co-expression analysis between miRNAs and genes was performed using Spearman’s rank correlation coefficient (ρ). Correlation with *p* < 0.05 was considered statistically significant. Correlation matrixes were generated with GraphPad Prism.

Differential gene expression analysis between normal ovaries and tumor tissues from serous OC patients was performed using the TNMplot web tool (www.tnmplot.com; accessed on 20 January 2024) integrating RNA-Seq data for normal and cancerous tissue from the Genotype–Tissue Expression (GTEx) and TCGA repositories, respectively [34]. Outliers were identified in each group according to the ROUT method with a 1% FDR using GraphPad Prism. The results were visualized by medians with box-and-whisker plots extending from the 25th to 75th percentiles. Statistical significance was assessed with an unpaired two-tailed Mann–Whitney test (non-normally distributed data).

### 2.10. Kaplan–Meier Survival Analysis for Ovarian Cancer Patients

Kaplan–Meier (KM) survival analysis was performed to assess prognostic values of mature miRNAs and their target genes in OC patients using a tumor online prognostic analysis platform (ToPP) (http://biostatistics.online/topp; accessed on 27 January 2024) with integrated data from TCGA project [35]. The analyses were restricted to HGSOC patients (grade III). For each gene, patients were split into two groups (low- and high-expression cohorts) according to the best cut-off value. A univariate module was employed to determine differences between groups regarding overall survival (OS), and progression-free interval (PFI). The difference between cohorts was characterized by the hazard ratio (HR) with 95% confidence intervals and log-rank *p* value.

### 2.11. Verification of Hub Genes’ Expression at Protein Level in Ovarian Cancer Patients

At the protein level, normal ovarian tissues and serous OC samples were compared for selected miRNA targets by immunohistochemistry (IHC) using the Human Protein Atlas database version 23.0 (HPA, www.proteinatlas.org; accessed on 30 January 2024) [36]. All antibodies fulfilled the enhanced validation principles. Protein expression was compared according to antibody staining intensity and fraction of stained cells based on HPA annotations: not detected, low, medium, or high.

### 2.12. Statistical Analysis

Statistical analysis was performed with GraphPad Prism version 10.1.2 for Windows (GraphPad Software, San Diego, CA, USA). Fold change of RT-qPCR expression data, calculated using the 2^−ΔΔCt^ method, was log-transformed to reduce skewness. The normality of data distribution was evaluated using either the Shapiro–Wilk or D’Agostino–Pearson test. Homogeneity of variance within groups was assessed using the Brown–Forsythe or Bartlett’s test. Statistical significance of differences among multiple groups was determined using ordinary one-way ANOVA followed by Tukey’s multiple comparison tests. Differences among groups were considered statistically significant at: * *p* < 0.05, ** *p* < 0.01, *** *p* < 0.001, **** *p* < 0.0001 (treatment vs. control); ^+^ *p* < 0.05, ^++^ *p* < 0.01, ^+++^ *p* < 0.001, ^++++^ *p* < 0.0001 (olaparib vs. combination with ATRi or CHK1i); ^#^ *p* < 0.05, ^##^ *p* < 0.01, ^###^ *p* < 0.001, ^####^ *p* < 0.0001 (ATRi or CHK1i vs. respective combinations with olaparib). Statistical significance for clinical data was assessed as described in the figure captions.

## 3. Results

### 3.1. Screening of Differentially Expressed miRNAs in Ovarian Cancer Cell Lines with Distinct Sensitivities to Olaparib

**Figure 1:**
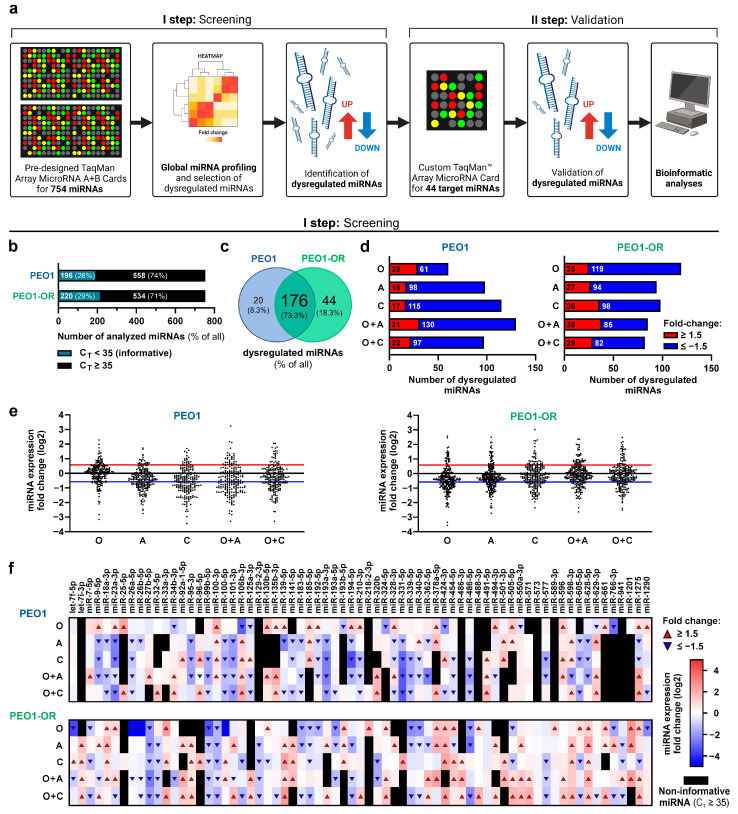
Two-step miRNA profiling strategy to screen and validate differentially expressed (DE) miRNAs in OC cell lines and miRNA screening results. (**a**) Workflow of the identification of DE miRNAs with pre-designed (I step) and custom (II step) TaqMan™ Array MicroRNA Cards covering 754 and 44 target miRNAs, respectively. (**b**) The number of informative miRNAs (C_T_ value < 35) detected in untreated PEO1 and PEO1-OR cells with pre-designed TaqMan™ Array MicroRNA Cards used for relative quantification of miRNA expression. (**c**) Venn diagram representing informative miRNAs (C_T_ value < 35) overlapping or unique for PEO1 and PEO1-OR cell lines detected with pre-designed TaqMan™ MicroRNA Array Cards. (**d**) The number of upregulated and downregulated miRNAs in PEO1 and PEO1-OR cells in response to olaparib (O) alone or combined with ATRi (A) or CHK1i (C) based on screening analysis. (**e**) Scatter dot plots representing a distribution of miRNA expression (logarithmized fold changes relative to untreated cells) in response to tested inhibitors in PEO1 and PEO1-OR cells. Dots above the red line and below the blue line indicate upregulated and downregulated miRNAs (absolute log_2_ of fold change ≥ 0.585), respectively. (**f**) Heatmap showing expression levels of 69 dysregulated miRNAs in PEO1 and PEO1-OR cells incubated with tested inhibitors. Red and blue triangles indicate upregulated and downregulated miRNA, respectively. Black rectangles indicate non-informative miRNAs in specific samples (C_T_ ≥ 35).

To establish the miRNA expression profile associated with acquired resistance to olaparib and understand the effects of combined treatments on miRNAs in HGSOC cell lines, we performed a large-scale miRNA differential expression analysis using a two-step approach (Figure 1a). A screening of 754 miRNAs was performed in PEO1 olaparib-sensitive and previously established PEO1-OR olaparib-resistant cells [17] using pre-designed TaqMan™ Array MicroRNA Cards. Based on the screening results, custom TaqMan™ Array MicroRNA Cards were designed and used for further validation of selected miRNAs in all OC cell lines.

The screening analysis revealed that 26% and 29% of all analyzed miRNAs were informative (C_T_ < 35) in untreated PEO1 and PEO1-OR cells, respectively (Figure 1b). Most of the informative miRNAs were shared between both cell lines (73%), whereas PEO1 and PEO1-OR cells expressed 8% and 18% unique miRNAs, respectively (Figure 1c). Numerous miRNAs were differentially expressed in OC cell lines treated with tested inhibitors indicating significant changes in miRNA profiles (Figure 1d,e). Specifically, a combination of olaparib with ATRi or CHK1i upregulated the expression of 38 and 29 miRNAs, whereas it downregulated 85 and 82 miRNAs in PEO1-OR cells, respectively (Figure 1d,e).

To select the most likely biologically relevant miRNAs, we focused on miRNAs that fulfilled any of the following criteria: miRNAs with the highest changes in expression levels, miRNAs present in PEO1-OR but undetected in PEO1 cells or vice versa, miRNAs induced or reduced by combinations of inhibitors compared with either inhibitor alone. Based on these assumptions, 69 dysregulated miRNAs were chosen (Figure 1f) for the bioinformatics selection of miRNAs for further expression validation.

Initial bioinformatic analysis was performed to prioritize and select the most critical dysregulated miRNAs (Table S2). One miRNA (hsa-miRNA-1201) is not currently annotated as a miRNA according to the latest miRBase database (v22) and it was rejected from further analyses. A preliminary miRNA–mRNA network was created with the web tool MIENTURNET [26] only for miRNAs with strong experimental evidence of interactions (Figure S1). Among all analyzed miRNAs, 11 miRNAs had no strong experimental evidence and were excluded from the analysis. The resulting miRNA–target interaction network topology for 57 miRNAs revealed that most miRNAs had a degree above or equal to three. However, 10 of the miRNAs were not connected with the working network and were assumed to be less biologically relevant (Figure S1).

Finally, the results of bioinformatic analyses and a literature review allowed us to select 44 dysregulated miRNAs (Table S3) from screening experiments for further validation in olaparib-sensitive (PEO1, PEO4) and olaparib-resistant (PEO1-OR) cell lines.

### 3.2. Comparison of miRNA Expression Patterns in Olaparib-Sensitive and Olaparib-Resistant HGSOC Cell Lines

**Figure 2:**
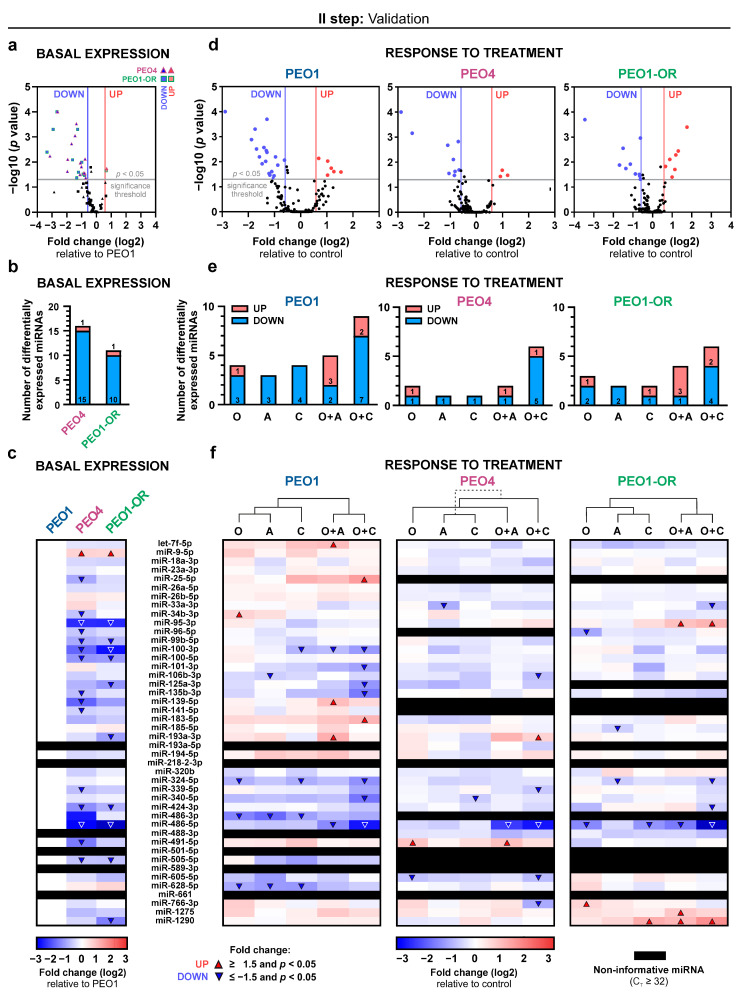
Overview of the miRNA basal expression in the absence of inhibitors and changes in miRNA levels in response to tested inhibitors in OC cell lines. (**a**,**d**) Volcano plots for miRNA expression: (**a**) basal expression in OC cells relative to PEO1 cells; (**d**) changes in expression in response to tested inhibitors or their combinations relative to untreated controls. DE miRNAs were identified according to the following criteria: absolute fold changes of expression ≥1.5 and *p* < 0.05 based on ordinary one-way ANOVA followed by multiple comparison tests. Significantly down- and upregulated miRNAs are highlighted with blue and red dots, respectively. Non-informative miRNAs with raw C_T_ values ≥ 32 in control cells are marked as black rectangles. (**b**,**e**) Bar charts representing the amount of significantly differentially expressed miRNAs: (**b**) basal expression relative to PEO1 cells; (**e**) changes in expression in response to tested inhibitors or their combinations relative to untreated controls. (**c**,**f**) Heatmaps for miRNA expression: (**c**) basal expression in OC cells relative to PEO1 cells; (**f**) expression changes in response to tested inhibitors or their combinations relative to untreated controls. Heatmaps were generated by a log transformation of the fold change data. Significantly (*p* < 0.05) down- and upregulated miRNAs (absolute fold change ≥ 1.5) are highlighted with blue and red triangles, respectively. Hierarchical clustering via heatmap was generated to visualize the clustering based on miRNA expression profiles associated with tested inhibitors.

We first compared the miRNA expression in olaparib-resistant PEO1-OR cells relative to PEO1 olaparib-sensitive cells to differentiate miRNA profiles associated with different sensitivities to olaparib (Figure 2). Additionally, we evaluated the miRNA expression in another olaparib-sensitive cell line, PEO4 (Figure 2) [7,17]. Selection and validation of stable expression of endogenous control genes for RT-qPCR data normalization are described in the Supplementary Data and presented in Figures S2 and S3.

Most of the miRNAs among the 44 analyzed (86%) were reliably detected and quantified in untreated HGSOC cell lines (Figure S4). Significantly differentially expressed (DE) miRNAs were identified based on statistical significance (*p* < 0.05) and absolute fold change ≥ 1.5 (i.e., log_2_ of fold change ≥ 0.585) (Figure 2a). Average fold change values for significantly dysregulated miRNA are presented in Table S4. The number of DE miRNAs in PEO1-OR cells and PEO4 cells compared to PEO1 cells amounted to 11 and 16 miRNAs, respectively. Differential miRNA expression was unbalanced, with 91% and 94% of all DE miRNAs being downregulated in PEO1-OR and PEO4 cell lines, respectively (Figure 2a,b). Comparison of miRNA profiles for all analyzed miRNAs showed substantial changes between PEO1 and PEO1-OR cells (Figure 2c). In PEO1-OR cells, miR-486-5p was the most downregulated (by 10.1-fold), whereas miR-9-5p was the most upregulated (by 1.6-fold) relative to PEO1 cells. The remaining DE miRNAs in PEO1-OR cells are as follows: miR-95-3p, miR-99b-5p, miR-100-3p, miR-100-5p, miR-125a-3p, miR-193a-3p, miR-424-3p, miR-505-5p, and miR-1290. Three of these miRNAs were uniquely downregulated in PEO1-OR cells only: miR-125a-3p, miR-193a-3p, and miR-1290 (Figure 2c).

On the whole, dysregulated expression of numerous miRNAs in the PEO1-OR cell line indicates highly likely altered post-translational regulation of gene expression associated with acquired resistance to olaparib in OC cells.

### 3.3. Differentially Expressed miRNAs Associated with Resensitization of PEO1-OR Cells to Olaparib with ATR/CHK1 Inhibitors

Previously we showed that ATR/CHK1 pathway inhibitors synergistically increase the cytotoxicity of olaparib in the olaparib-resistant HGSOC cell line by augmenting caspase-mediated apoptosis [7]. To identify miRNAs associated with resensitization of PEO1-OR to olaparib, we compared miRNA profiles in HGSOC cells treated for 48 h with olaparib alone or combined with ATRi or CHK1i. Most of the miRNAs were reliably detected and quantified in treated HGSOC cell lines (86% in PEO1, 75% in PEO1-OR, and 77% in PEO4 cells) (Figure 2). Expression of most miRNAs was unchanged (absolute fold change < 1.5 or *p* > 0.05) (Figure 2d). More DE miRNAs were identified as downregulated than upregulated in all HGSOC cells (Figure 2e). Average fold change values for significantly DE miRNAs are presented in Table S4. All results of RT-qPCR-based miRNA expression analysis in treated OC cell lines are presented in Figures S5–S7.

Cluster analysis between treatment groups revealed relatively distinct miRNA profiles between cells treated with single-agent inhibitors (O and A or C) and combined inhibitors (O + A, O + C) in PEO1 and PEO1-OR cell lines (Figure 2f). Co-clustering of olaparib combination groups indicates notable similarities at the miRNA level in PEO1-OR cells when olaparib cytotoxicity was augmented with the use of ATR/CHK1 pathway inhibitors.

**Figure 3:**
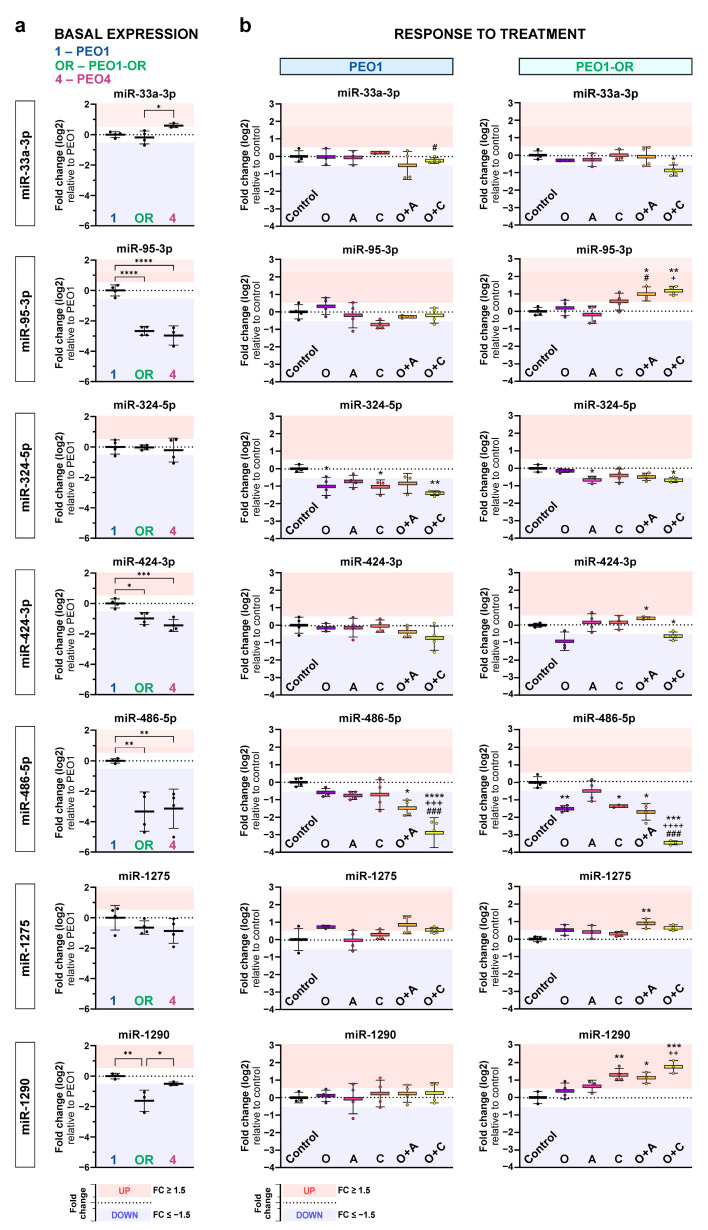
Differential expression analysis for miRNAs dysregulated in PEO1-OR cell line at basal levels (in the absence of inhibitors) and after treatments with tested combinations. (**a**) Basal miRNA expression in untreated PEO1-OR cells compared to PEO1 cells. (**b**) miRNA expression in PEO1-OR cells treated with olaparib (O), ATRi (A), CHK1i (C), or their combinations for 2 days. Levels of miRNA were determined via real-time qPCR and expressed as means of logarithmic fold change ± SD (*n* = 3–4). Statistical significance was assessed with ordinary one-way ANOVA followed by multiple comparison tests: * *p* < 0.05, ** *p* < 0.01, *** *p* < 0.001, **** *p* < 0.0001 (treatment vs. control); ^+^ *p* < 0.05, ^++^ *p* < 0.01, ^+++^ *p* < 0.001, ^++++^ *p* < 0.0001 (O vs. combination with A or C); ^#^ *p* < 0.05, ^###^ *p* < 0.001 (A or C vs. respective combinations with O). The red and blue areas indicate FC values for upregulated and downregulated miRNAs (absolute log_2_ of fold change ≥ 0.585), respectively.

In PEO1-OR cells incubated with olaparib alone, two miRNAs were downregulated (miR-96-5p, miR-486-5p), and one miRNA was upregulated (miR-766-3p) (Figure 2f). Combination treatments induced significant changes in the expression of seven miRNAs (four upregulated and three downregulated), some of which were overlapping in combination groups with either ATRi or CHK1i (Figure 2f). Four of these miRNAs (miR-33a-3p, miR-95-3p, miR-424-3p, miR-1275) were exclusively dysregulated in response to either combination in the PEO1-OR cell line, whereas they were unchanged in cells treated with single-agent inhibitors (Figure 2f). In PEO1-OR cells, four out of seven miRNAs dysregulated after olaparib combinations with ATRi or CHK1i were also changed at basal levels in the absence of inhibitors (miR-95-3p, miR-424-3p, miR-486-5p, and miR-1290) (Figure 2c,f). Interestingly, basal levels of miR-95-3p and miR-1290 were decreased in PEO1-OR cells in comparison with PEO1 cells (Figure 3a). However, the addition of ATRi or CHK1i to olaparib increased the expression of miR-95-3p and miR-1290 in PEO1-OR and appeared to partially restore miR-95-3p to the basal level in the absence of inhibitors observed in olaparib-sensitive cells (Figure 3a,b).

On the other hand, miR-424-3p and miR-486-5p were also negatively regulated in untreated PEO1-OR cells compared with PEO1 cells, but the inhibitor combinations further augmented the observed decrease in their expression levels. Interestingly, the addition of CHK1i to olaparib induced the decrease in miR-486-5p levels in both PEO1 and PEO1-OR cells (Figure 2f and Figure 3b).

Altogether, olaparib combined with the inhibition of the ATR/CHK1 pathway seemed to re-establish the expression of miR-95-3p and miR-1290 in PEO1-OR to the level observed in parental PEO1 cells. Moreover, PEO1 and PEO1-OR cells responded in the same manner to specific combination treatments concerning changes in miR-324-5p (O + C) and miR-486-5p (O + A and O + C) levels. Both types of alterations might be related to the resensitization of olaparib-resistant cells to olaparib.

### 3.4. Identification of miRNA–mRNA Regulatory Network, Enriched Pathways, and Biological Processes Related to Acquired Resistance to Olaparib in PEO1-OR Cells

**Figure 4:**
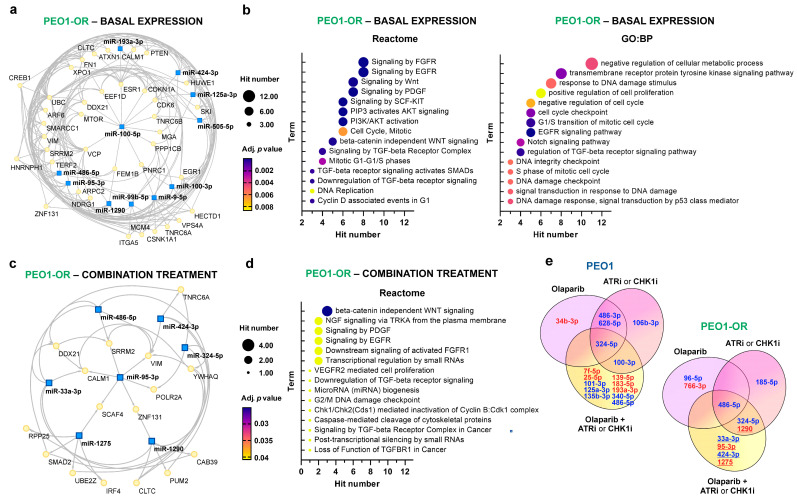
Network-based functional enrichment analyses of significantly differentially expressed (DE) miRNAs and their target genes in the PEO1-OR cell line. (**a**,**c**) The minimal miRNA–mRNA interaction networks in (**a**) untreated PEO1-OR cells and (**c**) PEO1-OR cells incubated with olaparib combinations. The blue square nodes represent miRNAs, and the yellow circular nodes represent target genes. (**b**,**d**) Enrichment terms visualized with bubble plots based on overrepresentation analysis for DE miRNA target genes in untreated PEO1-OR cells (**b**) and PEO1-OR cells incubated with olaparib combinations (**d**). The most significantly enriched functional annotations were selected following analysis with Reactome pathways and GO:BP databases. Terms were ranked by adjusted *p* value and number of target genes (hit). (**e**) Venn diagrams illustrating DE miRNAs in treated PEO1 and PEO1-OR cells. Significantly up- and downregulated miRNAs are highlighted with red and blue, respectively. Dysregulated miRNAs after combination treatments unique for PEO1-OR cells compared to PEO1 cells are underlined.

To further evaluate miRNAs likely connected with resistance to olaparib in HGSOC cells, we performed bioinformatics analyses of miRNA and target genes to explore the predicted biological functions of 11 DE miRNAs. The constructed minimal regulatory network (subnetwork) for dysregulated miRNAs illustrates 205 experimentally confirmed interactions between 11 miRNAs and 38 target genes (Figure 4a). Each miRNA was predicted to regulate between 7 and 21 target genes. Among all miRNAs, the highest degree centrality (DC) in the network was confirmed for miR-9-5p (DC = 21), miR-100-5p (DC = 12), and miR-125a-3p (DC = 12), representing their high importance in the interacting subnetwork.

Functional enrichment analyses indicated the top significantly enriched Reactome pathways in untreated PEO1-OR cells, including “signaling by fibroblast growth factor receptor (FGFR)”, “signaling by epidermal growth factor receptor (EGFR)”, and other Receptor tyrosine kinase (RTK) signaling pathways associated with growth factors (Figure 4b). Pathways involved in carcinogenesis, cell proliferation, cell migration, and cell cycle were also significantly enriched (Figure 4b). Importantly, the results provided by GO analysis were similar to enriched pathways detected with Reactome, suggesting the involvement of growth factor signaling (EGFR and TGF-β receptor) in decreased sensitivity of PEO1-OR cells to olaparib. Moreover, GO revealed significant biological processes associated with cell proliferation, cell cycle, DNA damage response, Notch signaling, and others (Figure 4b), partially in line with our previous studies [7,17]. Detailed results and target molecules involved in enriched terms for PEO1-OR cells are listed in Table S5.

Altogether, functional enrichment analysis identified pathways and processes that were likely regulated by DE miRNAs in PEO1-OR cells with acquired resistance to olaparib. Interestingly, a considerable number of their target genes were associated with the growth factor signaling pathways.

### 3.5. miRNA–mRNA Regulatory Network and Pathways Linked to Resensitization of PEO1-OR Cells to Olaparib with ATR/CHK1 Inhibitors

To unravel the potential role of dysregulated miRNAs and their target genes in PEO1-OR cells resensitized to olaparib in the presence of ATR/CHK1 pathway inhibitors, we constructed the miRNA–mRNA regulatory subnetwork and searched for enriched pathways and biological processes with overrepresentation analysis (Figure 4c,d). In PEO1-OR cells, five out of seven significantly DE miRNAs in response to olaparib combined with ATRi or CHK1i (miR-33a-3p, miR-95-3p, miR-424-3p, miR-1275, and miR-1290) were uniquely altered in olaparib-resistant cells but not in PEO1 cells (Figure 4e). The minimal miRNA–mRNA network was established with 23 nodes and 41 edges (experimentally confirmed interactions) for all seven dysregulated miRNAs in PEO1-OR (Figure 4c). Each miRNA was predicted to regulate between four and six target genes. Upregulated miR-95-3p was identified as a regulator of *POLR2A*, *CALM1*, *SRRM2*, *VIM*, *SCAF4*, and *ZNF131* in the subnetwork.

Then, we looked for pathways and biological processes that were enriched in target genes to elucidate functional insights into miRNAs’ regulatory properties in PEO1-OR cells treated with olaparib combinations (Figure 4d). The Reactome results revealed that β-catenin-independent WNT signaling was the most significantly enriched pathway associated with *TNRC6A*, *CALM1*, and *CLTC* target genes. We also found a few pathways associated with growth factor signaling by TGF-β, EGFR, FGFR1, NGF, VEGFR2, and PDGF to be significantly enriched in resensitized PEO1-OR cells (Figure 4d). Furthermore, pathways linked to apoptosis and cell cycle were found to be of significant enrichment in PEO1-OR cells. In agreement, our previous analysis indicated the role of caspase-mediated apoptosis [7] and abrogation of olaparib-induced G2/M arrest [17] in PEO1-OR cells in response to olaparib alone and combined with ATRi or CHK1i.

The GO:BP analysis showed no significantly enriched biological processes, likely due to the relatively small number of nodes in the network. Detailed results of analysis and target molecules involved in enriched terms for PEO1-OR cells are listed in Table S6. Overrepresentation analyses revealed some similarities and differences between PEO1 and PEO1-OR cells. Importantly, the most significantly enriched terms in PEO1 cells treated with either olaparib combination were also associated with various pathways and biological processes related to growth factors (Figure S8).

Overall, functional enrichment allowed us to determine pathways likely involved in the resensitization of PEO1-OR cells to olaparib in the presence of the ATR/CHK1 pathway inhibitors and focused our attention on growth factor signaling.

### 3.6. Hub Genes Associated with Olaparib Resistance and Resensitization of PEO1-OR Cells to Olaparib

**Figure 5:**
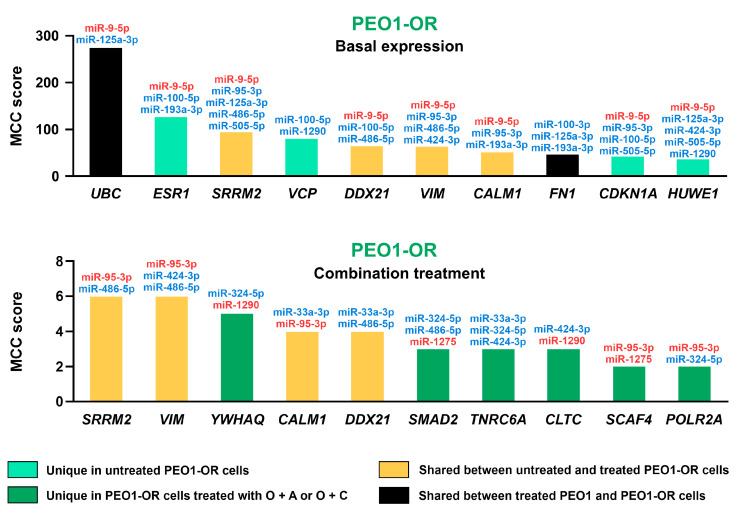
Hub genes associated with olaparib resistance and resensitization to olaparib with combination treatments in the PEO1-OR cell line. The top 10 hub genes were ranked (*x*-axis) based on the score (*y*-axis) calculated with the MCC algorithm using the cytoHubba plug-in in Cytoscape. Unique and shared genes are colored as described. Targeting miRNAs from the subnetwork are listed above bars for each hub gene from the PEO1-OR cell line and are highlighted with red (upregulated) or blue (downregulated) according to the results of relative quantity analysis.

To further screen out the core hub genes, we selected the top 10 genes with the highest connectivity in previously established subnetworks using the MCC algorithm in Cytoscape. Ranked hub genes and their connections with dysregulated miRNAs in PEO1 and PEO1-OR cells are illustrated in Figure 5.

From the analysis of hub genes, each was expected to be modulated by two to five DE miRNAs. Four genes seemed to be shared in untreated and combination-treated PEO1-OR cells (*SRRM2*, *DDX21*, *VIM*, *CALM1*) (Figure 5). As shown before in PEO1-OR cells, *CALM1* was associated with the top significantly enriched pathways including growth factor signaling by FGFR, EGFR, PDGF, and β-catenin-independent WNT signaling (Table S5). Another four genes were found to be uniquely associated with DE miRNAs in untreated PEO1-OR cells (*ESR1*, *VCP*, *CDKN1A*, and *HUWE1*) (Figure 5). *CDKN1A* was associated with most of the enriched pathways and processes in PEO1-OR cells including growth factor signaling (Table S5). Both *ESR1* and *CDKN1A* were linked with positive regulation of cell proliferation and cellular response to stress. Moreover, *VCP* and *CDKN1A* were found to be involved in response to DNA damage stimulus (Table S5). Six genes were uniquely linked with DE miRNAs in PEO1-OR cells resensitized to olaparib with ATRi or CHK1i (*YWHAQ*, *SMAD2*, *TNRC6A*, *CLTC*, *SCAF4*, and *POLR2A*) (Figure 5). These genes likely involved in resensitization to olaparib were associated with the following biological processes: beta-catenin-independent WNT signaling (*TNRC6A* and *CLTC*), G2/M DNA damage checkpoint (*YWHAQ*), loss of function of TGFβR1 in cancer (*SMAD2*), and transcriptional regulation by small RNAs (*TNRC6A* and *POLR2A*) (Table S5). Interestingly, no genes were shared between treated PEO1 and PEO1-OR cells (Figure 5). Experimentally validated targets of DE miRNAs from minimal networks are listed in Table S7.

Considering miRNAs, the MCC algorithm captured miR-9-5p, miR-125a-3p, and miR-100-5p as the top three miRNAs with the highest importance in untreated PEO1-OR cells (Table S8). Moreover, miR-95-3p, miR-486-5p, and miR-1290 were suggested as the three most essential miRNAs in PEO1-OR cells in response to olaparib combinations. Both miR-95-3p and miR-1290 were uniquely dysregulated after O + A or O + C treatments only in PEO1-OR cells (Figure 2b) and targeted five and two hub genes, respectively. Finally, key hub genes with the highest importance were obtained as signatures in the PEO1-OR cell line.

### 3.7. Olaparib Combined with ATR/CHK1 Inhibitors Dysregulates Proteins Involved in Growth Factor Signaling in PEO1-OR Cells

**Figure 6:**
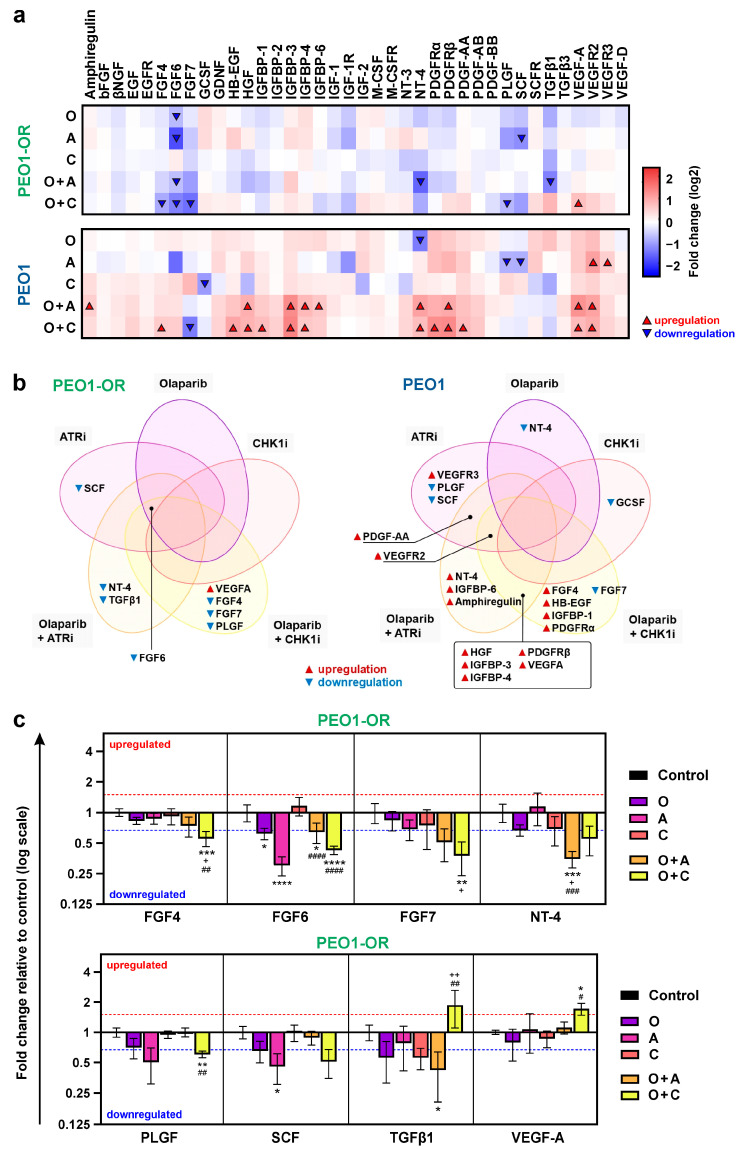
Olaparib combined with ATRi or CHK1i dysregulates the expression of growth factors (GFs) in OC cell lines. (**a**) Heatmaps for the expression of 41 GFs and their receptors in PEO1 and PEO1-OR cell lines. (**b**) Venn diagram for dysregulated GFs in PEO1 and PEO1-OR cell lines. (**c**) Results of semi-quantitative analysis with antibody microarrays for significantly dysregulated GFs in PEO1-OR cells (absolute fold change ≥ 1.5 and *p* < 0.05). Cells were incubated with inhibitors (O, A, C) or their combinations (O + A, O + C) for 2 days. Data are expressed as mean fold change ± SD (*n* = 4) on a logarithmized scale relative to untreated control cells. Statistical significance was assessed using ordinary one-way ANOVA followed by multiple comparison tests: * *p* < 0.05, ** *p* < 0.01, *** *p* < 0.001, **** *p* < 0.0001 (treatment vs. control); ^+^ *p* < 0.05, ^++^ *p* < 0.01 (O vs. combination with A or C); ^#^ *p* < 0.05, ^##^ *p* < 0.01, ^###^ *p* < 0.001, ^####^ *p* < 0.0001 (A or C vs. respective combinations with O).

Functional enrichment analyses indicated the involvement of growth factor (GF) signaling in olaparib resistance and restoration of OC cell sensitivity to olaparib with ATRi or CHK1i linked to dysregulated miRNAs. To test the biological relevance of bioinformatic analyses, we assessed the expression levels of 41 GFs and their receptors in PEO1 and PEO1-OR cells in the presence of tested inhibitors for 2 days using commercially available antibody microarrays (Figure 6 and Figure S9).

Heatmaps displayed relative quantities of 38 detected GFs in PEO1 and PEO1-OR cells shared between both cell lines (Figure 6a). Three GFs were not detected in neither PEO1 nor PEO1-OR cells (GM-CSF, TGFα and TGFβ2) (Figure S10). Single-agent inhibitors had an impact on the expression of GFs, significantly altering the levels by more than 1.5-fold (*p* < 0.05) of only three and six proteins in treated PEO1-OR and PEO1 cells, respectively (Figure 6a). In PEO1-OR cells, incubation with olaparib alone caused a significant downregulation of fibroblast growth factor (FGF6) by 1.6-fold.

Comparison of protein levels after incubation with olaparib combinations with ATRi or CHK1i revealed significant changes in the expression of 7 proteins in PEO1-OR cells and 15 proteins in PEO1 cells (Figure 6a). Dysregulated GFs, shared or unique for olaparib-sensitive and -resistant cell lines, are presented on Venn diagrams (Figure 6b). In response to combination treatments, most differentially expressed GFs were downregulated in PEO1-OR cells and upregulated in PEO1 cells. FGF4, FGF7, NT-4, PLGF, and TGFβ1 were exclusively downregulated in PEO1-OR cells in the presence of olaparib combined with either ATRi or CHK1i (Figure 6a,b).

The highest significant decrease in PEO1-OR cells was observed for NT-4 and FGF7, which were downregulated by 2.9-fold and 2.7-fold after olaparib treatment combined with ATRi and CHK1i, respectively (Figure 6c). VEGF-A was the only significantly upregulated GF in PEO1-OR cells (by 1.7-fold) after co-treatment with olaparib and the ATR/CHK1 pathway inhibitors, however, to a similar level as in PEO1 cells. Moreover, PEO1-OR cells abrogated combination-induced upregulation of numerous proteins associated with GFs and their receptors (amphiregulin, HB-EGF, HGF, IGFBP-1, IGFBP-3, IGFBP-4, IGFBP-6, PDGFRα, PDGFRβ, PDGF-AA, VEGF-A, and VEGFR2) which was observed in PEO1 cells (Figure 6a,b). Statistical analysis for significantly dysregulated GFs in PEO1 cells (Figure S9) and original representative images of antibody arrays are presented in the Supplementary Information (Figure S10).

Altogether, alterations in the expression of GFs were in line with the bioinformatic analyses indicating the association between GF signaling and miRNA dysregulation in PEO1-OR cells. Therefore, we speculated that different expression profiles could be the result of intrinsic characteristics of OC cell lines with distinct sensitivities to olaparib.

### 3.8. Differentially Expressed miRNAs and Target Genes Linked to Olaparib Resistance Predict Survival of Ovarian Cancer Patients

**Figure 7:**
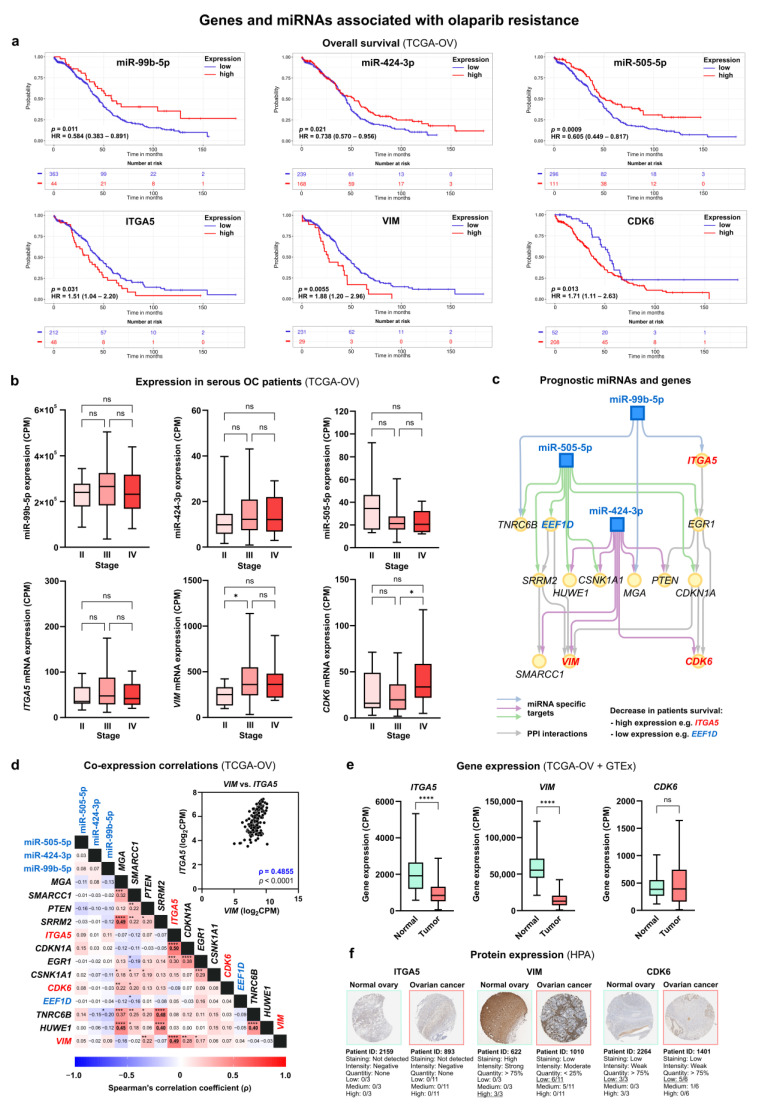
Differentially expressed miRNAs and target genes linked to olaparib resistance associated with survival in OC patients. (**a**) Interaction subnetwork between miRNAs associated with poor survival in serous OC cancer patients and target genes. The subnetwork originates from the minimal network for PEO1-OR cells under normal conditions linked to olaparib resistance. Nodes with two or more connections (colored arrows) were analyzed to highlight critical relationships. Expression levels of miRNAs and genes in OC patients significantly associated with survival are highlighted with blue (low expression) and red (high expression). (**b**) Stage-wise differential expression of miRNAs and genes associated with decreased survival in serous OC patients (TCGA-OV). Box plots show normalized CPM values extending from the 25th to 75th percentiles, lines dividing boxes represent medians, and the whiskers show the highest and lowest values after outlier removal within groups (FDR = 1%). Statistical significance was calculated using the Kruskal–Wallis test followed by Dunn’s multiple comparison test: * *p* < 0.05. (**c**) Kaplan–Meier (KM) plots display the relationship between miRNAs or genes and clinical endpoints in HGSOC patients (OS—overall survival, PFI—progression-free interval). Plots were generated with the ToPP web-based tool for HGSOC patients split into low- and high-expression groups using the best-performing threshold as a cut-off. Statistical significance between these two groups was calculated using the log-rank test: * *p* < 0.05. (**d**) Correlation matrix of miRNA and gene expression in serous OC patients (TCGA-OV). Correlations were computed using a two-tailed Spearman’s correlation test. Spearman’s rank correlation coefficient (ρ): 0–0.19 (no correlation), 0.20–0.39 (weak correlation), 0.40–0.59 (moderate correlation). Moderate correlations are highlighted with bold and underlined. Statistical significance: * *p* < 0.05, ** *p* < 0.01, *** *p* < 0.001, **** *p* < 0.0001. (**e**) Differential expression of genes associated with decreased survival in serous OC patients (TCGA-OV) between normal ovaries (GTEx) and OC (TCGA-OV). The analysis was performed using the RNA-seq data from the TNMplot web-based tool. Box plots show CPM values extending from the 25th to 75th percentiles, lines dividing boxes represent medians, and whiskers show the highest and lowest values after outlier removal within groups (FDR = 1%). Statistical significance was calculated using a two-tailed Mann–Whitney test: **** *p* < 0.0001. (**f**) Verification of protein expression for selected genes in normal ovaries and serous OC using the HPA database. Images show representative immunohistochemical staining for ITGA5 (HPA002642), VIM (CAB000080), and CDK6 (CAB004363).

**Figure 8:**
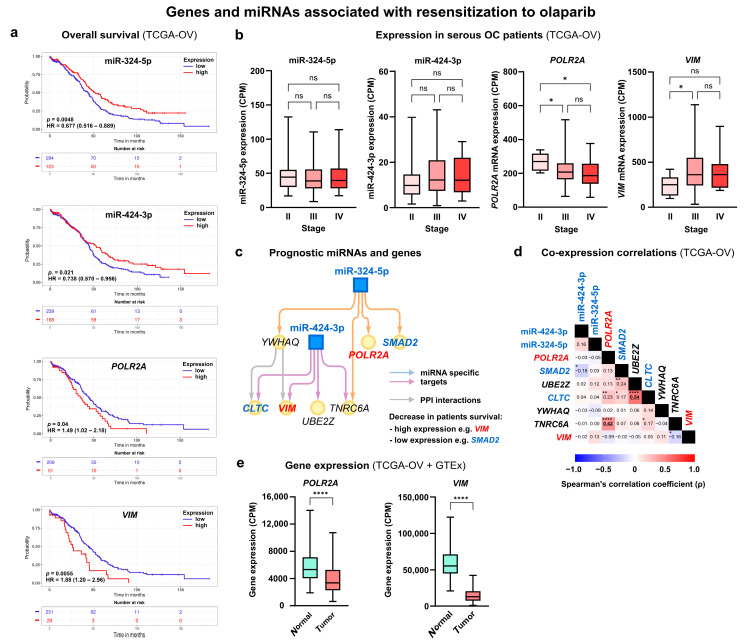
Differentially expressed miRNAs and target genes linked to resensitization to olaparib associated with survival in OC patients. (**a**) Interaction subnetwork between miRNAs associated with poor survival in serous OC cancer patients and target genes. The subnetwork originates from the minimal network for PEO1-OR cells treated with olaparib combined with ATR/CHK1 inhibitors linked to resensitization to olaparib. Expression levels of miRNAs and genes in OC patients significantly associated with survival are highlighted with blue (low expression) and red (high expression). (**b**) Stage-wise differential expression of miRNAs and genes associated with decreased survival in serous OC patients (TCGA-OV). Box plots show normalized CPM values extending from the 25th to 75th percentiles, lines dividing boxes represent medians, and the whiskers show the highest and lowest values after outlier removal within groups (FDR = 1%). Statistical significance was calculated using the Kruskal–Wallis test followed by Dunn’s multiple comparison test: * *p* < 0.05. (**c**) Kaplan–Meier (KM) plots showing the relationship between miRNAs or genes and clinical endpoints in HGSOC patients (OS—overall survival, PFI—progression-free interval). Plots were generated with the ToPP web-based tool for HGSOC patients split into low- and high-expression groups using the best-performing threshold as a cut-off. Statistical significance between these two groups was calculated using the log-rank test: * *p* < 0.05. (**d**) Correlation matrix of miRNA and gene expression in serous OC patients (TCGA-OV). Correlations were computed for every pair of datasets using a two-tailed Spearman’s correlation test. Spearman’s rank correlation coefficient (ρ): 0–0.19 (no correlation), 0.20–0.39 (weak correlation), 0.40–0.59 (moderate correlation). Moderate correlations are highlighted with bold and underlined. Statistical significance: * *p* < 0.05, ** *p* < 0.01, **** *p* < 0.0001. (**e**) Differential expression of genes associated with decreased survival in serous OC patients (TCGA-OV) between normal ovaries (GTEx) and OC (TCGA-OV). The analysis was performed using the RNA-seq data from the TNMplot web-based tool integrating the data for normal and cancerous tissues. Box plots show CPM values extending from the 25th to 75th percentiles, the line dividing the box represents the median, and the whiskers show the highest and lowest values after outlier removal within groups (FDR = 1%). Statistical significance was calculated using a two-tailed Mann–Whitney test: **** *p* < 0.0001.

After overlapping miRNAs dysregulated in untreated PEO1-OR cells and in response to olaparib combinations (O + A or O + C), 14 DE miRNAs were selected for further analysis using clinical data from TCGA for stage II–IV serous OC patients (Table 1). Four miRNAs were common (miR-95-3p, miR-424-3p, miR-486-5p, and miR-1290), seven were unique in untreated PEO1-OR cells (miR-9-5p, miR-99b-5p, miR-100-3p, miR-100-5p, miR-125a-3p, miR-193a-3p, and miR-505-5p), and three were unique for treated PEO1-OR cells (miR-33a-3p, miR-324-5p, and miR-1275). Examining these miRNAs in clinical samples involved assessing their association with patient survival and expression in serous OC patient samples regarding olaparib resistance (Figure 7) and resensitization to olaparib (Figure 8).

Firstly, to highlight miRNAs that likely possess biological functions in vivo, miRNA abundance was assessed in OC samples using counts per million (CPM) values. We focused on relatively abundant miRNAs determined with a pre-defined cut-off (CPM ≥ 10 in ≥50% samples) resulting in eight miRNAs for further analysis (miR-9-5p, miR-99b-5p, miR-100-5p, miR-125a-3p, miR-324-5p, miR-424-3p, miR-486-5p, and miR-505-5p) (Table 1).

We initially focused on miRNAs associated with resistance to olaparib due to their dysregulation in untreated PEO1-OR cells relative to PEO1 cells. For clinical relevance, we used the ToPP web tool for Kaplan–Meier (KM) survival analysis on filtered HGSOC data from TCGA-OV. Notably, three of seven highly abundant miRNAs (miR-99b-5p, miR-424-3p, and miR-505-5p) showed significant prognostic value for both overall survival (OS) and progression-free intervals (PFIs) (Table 1). Low levels of miR-99b-5p, miR-424-3p, and miR-505-5p were linked to significantly worse OS times (Figure 7a). Notably, decreased expression of these miRNAs was observed in PEO1-OR cells compared to PEO1 cells (Figure 2c). KM plots for all analyzed miRNAs are presented in Figure S11. Stage-wise differential expression analysis showed no significant differences in the expression of three selected miRNAs across stages II, III, and IV of serous OC (Figure 7b). Stable expression of mature miRNAs over higher stages suggested their potential role in disease progression.

Subsequently, we created an interaction subnetwork for miR-99b-5p, miR-424-3p, and miR-505-5p and 13 target genes with two or more connections within the network (Figure 7c), reducing the previous network for untreated PEO1-OR cells (Figure 4a).

Genes targeted by prognostic miRNAs from the network are listed in Table 2. Notably, target genes regulated by prognostic miRNAs were associated with 80% of the previously established pathways and biological processes enriched in PEO1-OR cells, such as signaling by GF, signaling by Wnt, and cell cycle regulation (Table S5).

Based on survival data, four genes (*EEF1D*, *ITGA5*, *VIM*, and *CDK6*) were associated with both OS and PFI (Figure 7a, Figures S12 and S13). Considering that low expression of selected miRNAs was linked to worse survival, we looked for target genes with high expression showing unfavorable OS and PFI. HGSOC patients with high *ITGA5*, *VIM*, and *CDK6* showed significantly decreased survival represented by HR of 1.51 (*p* = 0.031), 1.88 (*p* = 0.0055), and 1.71 (*p* = 0.013) (Figure 7a). Interestingly, the *CDK6* gene was associated with enriched pathways regulating the cell cycle, previously linked to olaparib resistance in PEO1-OR cells (Table S5). Stage-wise differential expression revealed upregulation of the *VIM* gene in stage III compared to stage II and upregulation of the *CDK6* gene in stage IV compared to stage III in serous OC patients (Figure 7b). These data indicate that *VIM* and *CDK6* expression may be potentially linked to tumor progression.

Next, correlation analysis revealed a positive moderate correlation between mRNA expression of *ITGA5* and *VIM* genes (ρ = 0.49, *p* < 0.0001) in serous OC patients (Figure 7d). A comparison of gene expression in normal ovaries and tumor tissue revealed decreased mRNA levels of *ITGA5* and *VIM* in OC patients (Figure 7e). Verification of gene expression at the protein level using the HPA database showed that VIM is downregulated in most OC samples relative to normal tissue (Figure 7f) which is in line with the differential mRNA expression. Immunohistochemical images of ITGA5 showed weak staining in both normal and tumor tissue (Figure 7f), however, ITGA5 levels were annotated as “not detected” in the HPA database and rejected from interpretation.

Our analysis revealed key miRNAs associated with olaparib resistance in vitro that are also dysregulated in serous OC patients. Given that downregulated miRNAs (miR-99b-5p, miR-424-3p, and miR-505-5p) and upregulated target genes (*VIM*, *ITGA5*, *CDK6*) are also linked to worse survival of women with OC, our findings provide a theoretical foundation for exploring the link between miRNAs and resistance to olaparib in vivo.

### 3.9. Prognostic Roles of Differentially Expressed miRNAs and Target Genes Associated with Resensitization to Olaparib for Ovarian Cancer Patients

Additionally, we assessed the relevance of three miRNAs (miR-324-5p, miR-424-3p, and miR-486-5p) linked to olaparib resensitization in PEO1-OR cells based on clinical data for serous OC patients, focusing on only highly abundant miRNAs in clinical samples (Table 2). KM survival analysis indicated that low expression of miR-324-5p and miR-424-3p predicted unfavorable OS and PFI (Table 2). KM plots for OS are presented in Figure 8a. Downregulation of miR-324-5p and miR-424-3p occurred in PEO1-OR cells in response to olaparib combinations with ATRi or CHK1i (Figure 2c). Interestingly, both miRNAs exhibited unchanged expression across stages II, III, and IV in serous OC patients, indicating their potential role throughout tumor development (Figure 8b).

Using miRNAs with significant prognostic value, we created an interaction subnetwork for miR-324-5p and miR-424-3p and seven target genes (Figure 8c), reducing the previous network established for PEO1-OR cells treated with olaparib combinations (Figure 4c). Clinical prognostic significance was found for *POLR2A*, *VIM*, *CLTC*, and *SMAD2* genes (Table 2). These genes were previously established in pathways involved in the resensitization of PEO1-OR cells to olaparib (Table S6). Connecting unfavorable survival prognosis with low miRNA expression, we identified genes associated with worse survival in high-expression compared to low-expression OC cohorts. Serous OC patients with increased *VIM* and *POLR2A* had worse OS and PFI compared to low-expression groups (Figure 8a). Overexpression of the *VIM* gene was linked to the worst OS time (HR = 1.88, *p* = 0.0055). Both *VIM* and *POLR2A* genes were associated with pathways enriched in resensitized PEO1-OR cells through regulation of caspase-mediated apoptosis and miRNA biogenesis (Table S6).

Considering genes associated with unfavorable survival, stage-wise differential expression analysis indicated downregulation of *POLR2A* in stages III and IV compared to stage II in OC patients and dysregulation of *VIM* (Figure 8b). Correlation analysis revealed a significant relationship between a few genes, but none were found for selected prognostic genes (Figure 8d). However, *POLR2A* showed a positive association with *TNRC6A* at the transcriptional level (ρ = 0.42, *p* < 0.0001). *POLR2A* and *VIM* were significantly downregulated in OC tissue in comparison with normal ovaries (Figure 8e). The HPA provided no data for POLR2A at the protein level which impeded validation of the expression.

Overall, miR-324-5p and miR-424-3p were significantly related to the survival of OC patients and antitumor response in resensitized PEO1-OR cells. Therefore, the abovementioned miRNAs and potentially their target genes provide promising predictive information to understand the reversal of olaparib resistance with the ATR/CHK1 pathway inhibitors in the context of serous OC.

## 4. Discussion

Olaparib exhibits significant clinical benefits in newly diagnosed and recurrent HGSOC patients, especially as maintenance therapy that prolongs overall survival in platinum-sensitive OC patients with *BRCA1/2* mutations [37]. Interestingly, responsiveness to PARPi is usually closely associated with platinum sensitivity [37,38]. However, resistance to olaparib can develop over time in some patients, necessitating a critical understanding of associated mechanisms. Indeed, multifactorial mechanisms of resistance to PARPi and platinum analogs were demonstrated to be at least partially interrelated in OC cells [8,39]. Recent studies have demonstrated that modulators of DNA damage response, including the ATR/CHK1 pathway inhibitors, can resensitize OC cells both in vitro and in vivo [10,39,40]. Despite extensive research on resistance mechanisms, including epigenetic silencing of gene expression [41], there is still a need to better understand OC desensitization to olaparib, particularly at the post-transcriptional level. The novelty of the study is the use of the *BRCA2*^MUT^ HGSOC cell line PEO1-OR with acquired resistance to olaparib to understand mechanisms linked to miRNA regulatory properties responsible for distinct sensitivities to olaparib alone or combined with the ATR/CHK1 pathway inhibitors. As olaparib-sensitive HGSOC models [7,17], we employed PEO1 and PEO4 cells, which were established from the same patient at the first and the second relapse following chemotherapy and represent a clinical progression of OC [18,19]. Moreover, the role of DE miRNAs and their target genes was examined in the context of serous OC patient survival to select essential signature molecules.

Considering olaparib resistance, miRNA profiling of PEO1-OR cells compared to parental PEO1 cells in the absence of inhibitors revealed 11 DE miRNAs with absolute fold change ≥ 1.5 and *p* < 0.05 (miR-9-5p, miR-95-3p, miR-99b-5p, miR-100-3p, miR-100-5p, miR-125a-3p, miR-193a-3p, miR-424-3p, miR-486-5p, miR-505-5p, miR-1290). Most of them were significantly downregulated, except for upregulated miR-9-5p. The expression profile of miRNAs was also established in PEO4 cells. These cells exhibit a sensitivity to olaparib that is more similar to PEO1-OR cells after short-term incubation with olaparib (2 days), whereas after long-term incubation (5 days), their sensitivity becomes evident, similar to that of PEO1 cells [7,17]. While PEO4 cells are indeed olaparib-sensitive, it takes more time for olaparib to manifest its cytotoxic activity in these cells compared to PEO1 cells [7,17]. Therefore, we consider the drug-induced changes observed in PEO4 cells after 48 h, such as the deregulation of miRNAs, as early cellular events associated with the antitumor activity. Consequently, we hypothesize that PEO4 cells would share some DE miRNAs with both PEO1 and PEO1-OR cells. Interestingly, eight DE miRNAs were common in PEO1-OR cells and PEO4 cells, and we presumed that these miRNAs were likely associated with short-term desensitization to olaparib when both cell lines possess a phenotype of decreased sensitivity. Moreover, three DE miRNAs (miR-125a-3p, miR-193a-3p, miR-1290) were unique for PEO1-OR cells, which distinguished their characteristics of acquired long-term resistance.

Some downregulated miRNAs identified in our work were also reported in studies deciphering miRNA profiles in solid tumor samples, serum, and exosomes from serous OC cells, including miR-99b (GSE76449), miR-100-5p (GSE83693), miR-125a-3p (GSE106817), miR-193a-3p (GSE83693), miR-424-3p (GSE47841), miR-486-5p (GSE47841), and miR-505-5p (GSE76449). The study by Nam et al. demonstrated the downregulation of miR-100-5p in paired primary and recurrent HGSOC samples obtained from patients after cytoreductive surgeries compared to normal ovarian tissue [42]. Overall, we indicate that all revealed DE miRNAs contribute to the olaparib-resistant phenotype of PEO1-OR HGSOC cells in vitro.

The created minimal subnetwork with protein–protein interactions maximally connected 11 DE miRNAs in PEO1-OR cells with 38 target genes revealing essential interactions. Functional enrichment analyses indicated the potential involvement of a few interconnected biological processes and pathways in acquired resistance to olaparib. We showed that genes targeted with DE miRNAs were significantly associated with growth factor (GF) signaling, particularly signaling by fibroblast GF receptor (FGFR), epidermal GF receptor (EGFR), platelet-derived GF (PDGF), and stem cell factor (SCF)-KIT. Recently, Nicholson et al. revealed a link between FGFR signaling, DNA damage response, and resistance to cisplatin in human OC cell lines [43]. Increased activation of EGFR induced by FGFR3 overexpression was associated with decreased sensitivity to cisplatin in serous OC cells [44]. Here, the downregulation of transforming GF beta receptor (TGFβR) signaling was predicted as a significant change in signal transduction in PEO1-OR cells. The tumorigenic role of TGFβ has been extensively studied in OC cells regarding its association with epithelial-to-mesenchymal transition (EMT) [45]. Resistance to olaparib promoted by the TGFβ pathway has been described in various tumors [46,47], however, findings from OC are lacking. From enriched pathways linked to GF signaling, we identified a set of eleven key genes (*UBC*, *TNRC6A*, *TNRC6B*, *MTOR*, *CREB1*, *CDKN1A*, *CALM1*, *PTEN*, *XPO1*, *PPP1CB*, *SKI*). In the context of resistance, silencing of cyclin-dependent kinase inhibitor 1A (CDKN1A) with miRNAs was previously suggested to promote cisplatin resistance in OC cells in vivo [48]. Additionally, we identified phosphatidylinositol 3-kinase (PI3K)/AKT signaling to be significantly enriched in PEO1-OR cells. Experiments employing cisplatin-resistant OC cell lines showed that activation of the PI3K/AKT pathway desensitized cells to chemotherapy [49]. More recently, Xu et al. proposed that a combination of AKT inhibitor with olaparib slowed down tumor growth in a patient-derived xenograft (PDX) model of recurrent platinum-resistant OC with prior PARPi therapy [50].

The functional enrichment analysis also indicated GF signaling’s role in decreased sensitivity of the PEO1-OR cell line to olaparib. Moreover, we found that PEO1-OR cells were enriched in biological processes related to cell cycle regulation and DNA integrity checkpoints, aligning with our previous findings of abrogated olaparib-induced G2/M arrest [17]. Here, we revealed that genes implicated in progression through the cell cycle (*MCM4*, *CDK6*, *CDKN1A*, *FEM1B*, *NDRG1*, *PTEN*, and *UBC*) may be targeted by DE miRNAs in PEO1-OR cells. Phosphatase and tensin homolog (PTEN) is a protein involved in regulating the PI3K/AKT pathway. The study by Selvendiran et al. demonstrated that upregulation of PTEN prompted G2/M arrest and apoptosis in cisplatin-resistant OC cells, however, the association with resistance to olaparib requires further investigation [51]. Altogether, we identified target genes of particular interest in the context of resistance to olaparib in our cell line model of HGSOC for potential further investigation to gain more insights into their role in vitro and in vivo. 

To assess the biological relevance of our in vitro cell line data, we performed survival analysis for selected DE miRNAs and target genes in HGSOC patients using data from TCGA-OV. Based on recommendations of TCGA Research Network, clinical survival outcome for OC patients was evaluated using overall survival (OS) and progression-free intervals (PFIs) [52]. Next, we reconstructed a minimal miRNA–mRNA network associated with olaparib resistance, prioritizing prognostic miRNAs and their target genes. Kaplan–Meier analyses revealed that three miRNAs (miR-99b-5p, miR-424-3p, and miR-505-5p), downregulated in untreated PEO1-OR cells, were significantly associated with shorter OS and PFI in low-expression cohorts of HGSOC patients. A previous study demonstrated miR-99b-5p downregulation in the plasma exosomes of treatment-naïve OC patients compared to healthy individuals [53]. Additionally, we revealed that OC patients with high levels of integrin α5 (ITGA5), a target for miR-99b-5p, showed poorer survival compared to a low-expression cohort. ITGA5 is a transmembrane protein reported to promote metastasis in HGSOC cells [54], which is in line with unfavorable survival. In vivo experiments revealed that loss of ITGA5 inhibits the growth of OC xenografts [54]. A few studies examined the effect of miR-424-3p on proliferation, migration, and apoptosis in OC. A recent study suggested that miR-424-3p increases the sensitivity of OC cell lines to cisplatin by downregulating the antiapoptotic protein galectin-3 in vitro [55]. This indicates that the downregulation of miR-424-3p in PEO1-OR cells may be partially responsible for the olaparib-resistant phenotype. Moreover, we showed that high levels of genes encoding vimentin (*VIM*) and cyclin-dependent kinase 6 (*CDK6*), both targeted by miR-424-3p, were linked to shorter OS and PFI. Indeed, the upregulation of vimentin was demonstrated to induce EMT, cell growth, invasion, and chemoresistance in both OC cell lines and xenograft models of OC [56,57]. Moreover, in vitro and in vivo experiments highlighted that increased levels of CDK6 were linked to upregulation of ATR kinase and decreased cell death in OC treated with cisplatin [58]. Altogether, we revealed important miRNA–mRNA interactions and processes associated with decreased sensitivity to olaparib in *BRCA2*^MUT^ OC cells in vitro.

Despite initial effectiveness, the widespread use of olaparib involves an increasing number of patients with acquired resistance and a lack of further approved therapy options [39]. Over recent years, combinations of olaparib with the ATR/CHK1 pathway inhibitors showed promising antitumor activity in OC cells [9,10,40]. The results of the CAPRI study revealed that patients with recurrent platinum-sensitive *BRCA1/2*^MUT^ HGSOC, who had progressed upon prior PARPi, obtained promising partial responses to olaparib combined with the ATRi ceralasertib [10]. Our previous work demonstrated that inhibitors of ATR and CHK1 kinases exerted synergistic cytotoxic activity with olaparib in PEO1-OR cells [7]. Hence, the second main goal of our study was to investigate the miRNA profile of HGSOC cells resensitized to olaparib with the ATR/CHK1 pathway inhibitors.

A few previous studies analyzed the roles of specific miRNAs in the cytotoxicity of olaparib in OC cells, however, many of them utilized cell lines that do not possess features of HGSOC. The recent study suggested that overexpression of miR-200c sensitized the *BRCA1*^WT^ SKOV-3 cells to olaparib by increasing apoptosis [59]. In another work, it was demonstrated that targeted inhibition of cyclin D1 by miR-20b increased the cytotoxicity of olaparib in SKOV-3 cells and cell-line-derived xenograft models of OC [60].

Here, we identified six dysregulated mature miRNAs that have so far not been mentioned in the context of overcoming resistance to olaparib in HGSOC cells using the ATR/CHK1 pathway inhibitors. Resensitization of PEO1-OR cells was associated with upregulation of miR-95b-3p and miR-1290 along with downregulation of miR-33a-3p, miR-324-5p, miR-424-3p, and miR-486-5p. Most of these DE miRNAs were unique for PEO1-OR cells compared to olaparib-sensitive cells. Interestingly, the addition of ATRi or CHK1i to olaparib further augmented a decrease in miR-424-3p and miR-486-5p observed in untreated PEO1-OR cells. Through analysis of miRNA–gene interactions, we found that these six miRNAs could build highly connected linkage with 17 experimentally validated target genes. Network-based approaches revealed that several pathways might be dysregulated by target genes involved in overcoming resistance to olaparib. Overrepresentation analysis found that resensitized PEO1-OR cells were mainly enriched in four groups of processes: GF signaling, apoptosis, cell cycle checkpoints, and gene silencing by RNA.

In this study, GF signaling represented a new process of particular interest associated with TGFβR1, EGFR, PDGF, and nerve growth factor (NGF). SMAD2 gene, targeted by three DE miRNAs, was found to be enriched in the pathways controlled by TGFβ receptors. Findings by Roberts et al. revealed that treatment with TGFβ induced EMT and downregulated homologous recombination repair protein, resulting in resensitization of HGSOC cells to olaparib in vitro [61]. Here, we showed that enriched signaling by FGFR1, EGFR, PDGF as well as VEGFR2 mediated cell proliferation was associated with genes encoding trinucleotide repeat-containing gene 6A protein (TNRC6A) and calmodulin 1 (CALM1), targeted by three and two DE miRNAs, respectively. Calmodulin is a calcium-dependent protein that binds and regulates the activity of EGFR [62]. EGFR and VEGFR2 are two membrane RTK proteins frequently upregulated in OC [63]. Interestingly, therapies combining platinum with VEGF/VEGFR inhibitors showed higher efficacy than monotherapies in platinum-resistant OC patients [64]. Moreover, our bioinformatic analysis confirmed that the G2/M DNA damage checkpoint may be involved in the resensitization of PEO1-OR cells, which agrees with our previous studies [17]. Here, we predicted that the YWHAQ gene, targeted by dysregulated miR-324-5p and miR-1290, may play a critical role in this process. YWHAQ, also known as 14-3-3σ, is a protein involved in the inhibition of G2/M progression [65] and contributes to the pathogenesis of epithelial OC [66]. Taken together, we predicted that a few biological processes might be implicated in the resensitization of PEO1-OR cells to olaparib using ATRi or CHK1i.

Subsequently, we validated the biological possibility of the findings concerning GF signaling through downstream expression analyses for 41 human GFs and their receptors. PEO1-OR cells abrogated upregulation of 13 GFs observed in PEO1 cells treated with olaparib combined with the ATR/CHK1 pathway inhibitors. This suggested different responses of olaparib-sensitive and olaparib-resistant cells to combination treatments in the context of GF signaling. We uncovered altered expression of five GFs uniquely in PEO1-OR cells after combination treatments (FGF4, FGF6, NT-4, PLGF, and TGFβ1). FGF4, neurotrophin-4 (NT-4), and TGFβ1 were significantly downregulated and VEGF-A upregulated only in the presence of combined inhibitors. Previous research has shown that overexpression of FGF4 in fibroblast mixed cancer stem-like cells isolated from OC increases their sphere-forming capacity, however, knockdown of *FGF4* can abrogate it [67]. In epithelial OC cells, the upregulation of VEGF-A via PARP1 can promote angiogenesis [68]. Moreover, OC cells overexpressing TGFβ1 exhibited decreased sensitivity to cisplatin [69]. Overall, dysregulation of GF expression in desensitized PEO1-OR cells confirmed our predictions from bioinformatic analyses. Accordingly, we pointed out that downregulation of FGF4, VEGF-A, TGFβ1, and possibly NT-4 could play an important role in the resensitization of PEO1-OR cells to olaparib.

Finally, we investigated the role of DE miRNAs in combination-treated PEO1-OR cells and their target genes in the survival of HGSOC patients. Our study revealed a significant association between low levels of miR-324-5p and miR-424-3p, both downregulated in PEO1-OR cells, and poor OS and PFI times in OC patients. We investigated a set of experimentally validated targets of miR-324-5p and miR-424-3p. Importantly, low levels of two targets, vimentin and POLR2A, displayed an inverse association with OC survival compared to both miRNAs. Vimentin has been shown to promote metastatic progression by EMT in solid tumors [70]. The recent study showed that OC cells resistant to olaparib upregulate vimentin expression at the mRNA level irrespective of *BRCA1* status. Moreover, the downregulation of vimentin was associated with the inhibition of PARP1, resulting in decreased viability of OC cells in vitro [71]. Interestingly, our previous study highlighted that PEO1-OR cells incubated with olaparib and ATR/CHK1 pathway inhibitors also downregulate PARP1 [7]. POLR2A is a DNA-dependent RNA polymerase essential for cell survival. In vivo studies demonstrated that POLR2A expression level was higher in PDX models of cisplatin-resistant OC compared to sensitive cells [72].

We acknowledge that our study has several limitations. Here we experimentally confirmed dysregulation of miRNA expression in olaparib-resistant cells, however, a complex interplay among miRNAs, mRNAs, and other unexplored functional elements including long non-coding RNA requires further investigation. Furthermore, functional assays with miRNA mimics and inhibitors might support our findings concerning the role of significantly dysregulated miRNAs in acquired resistance and resensitization to olaparib. Nonetheless, this study highlights a new molecular basis associated with miRNAs for revealing mechanisms of olaparib resistance and overcoming it with the ATR/CHK1 pathway inhibitors.

## 5. Conclusions

In conclusion, we described dysregulated miRNAs and their interactions with essential target genes associated with the survival of serous OC patients. Placing these results in the context of prior studies, our findings highlight the altered miRNA profile of PARPi-resistant HGSOC cells with restored BRCA2 in vitro. This may be especially important in the context of predicting response to olaparib in OC patients. Our data indicate that GF signaling may play an important role in acquired resistance and resensitization to olaparib. Further examination of the linkage between miRNAs and response to olaparib alone or combined with the ATR/CHK1 inhibitors in vivo may shed light on the rationale for combination therapy in HGSOC patients. Importantly, this cell-line-based miRNA profiling study could serve as a platform for further research on olaparib resistance and resensitization in ovarian cancer.

## Figures and Tables

**Table 1:** Overview of differentially expressed miRNAs in PEO1-OR cell line selected for analysis based on their expression abundancy and prognostic value in OC patient samples from TCGA database. Significant clinical endpoints are marked with bold (log-rank *p* < 0.05).

miRNAs	Expression in PEO1-OR Cells		Percentage of Samples with CPM ≥ 10 in TCGA-OV *	OS in Serous Ovarian Cancer Patients (High vs. Low Expression)		PFI in Serous Ovarian Cancer Patients (High vs. Low Expression)	
	Standard Conditions	Combination Treatments		HR	Log-Rank *p*	HR	Log-Rank *p*
miR-9-5p	▲		89%	0.84	0.31	1.22	0.11
miR-99b-5p	▼		100%	**0.58**	**0.011**	**0.539**	**0.0025**
miR-100-3p	▼		0%	n/a	n/a	n/a	n/a
miR-100-5p	▼		100%	1.18	0.19	**1.36**	**0.0088**
miR-125a-3p	▼		99%	**1.37**	**0.039**	1.17	0.22
miR-193a-3p	▼		1%	n/a	n/a	n/a	n/a
miR-505-5p	▼		95%	**0.61**	**0.0009**	0.793	0.065
miR-95-3p	▼	▲	4%	n/a	n/a	n/a	n/a
miR-424-3p	▼	▼	63%	**0.74**	**0.021**	**0.787**	**0.044**
miR-486-5p	▼	▼	100%	1.13	0.36	1.24	0.068
miR-1290	▼	▲	0%	n/a	n/a	n/a	n/a
miR-33a-3p		▼	0%	n/a	n/a	n/a	n/a
miR-324-5p		▼	99%	**0.68**	**0.0048**	0.821	0.094
miR-1275		▼	11%	n/a	n/a	n/a	n/a

* miRNAs with CPM ≥ 10 in ≥50% samples from TCGA-OV were subjected to further analyses, whereas miRNAs with CPM < 10 in ≥50% samples were rejected and designated as not applicable (n/a). **CPM**—counts per million, **HR**—hazard ratio, **OS**—overall survival, **PFI**—progression-free interval, ▲—upregulated in PEO1-OR cells, ▼—downregulated in PEO1-OR cells.

**Table 2:** Overview of genes targeted by DE miRNAs associated with survival selected for analysis based on their expression abundancy and prognostic value in OC patient samples from TCGA database. Significant clinical endpoints are marked with bold (log-rank *p* < 0.05).

Gene	Percentage of Samples with CPM ≥ 10 in TCGA-OV *	OS in Serous OC Patients (High vs. Low Expression Cohort)		PFI in Serous OC Patients (High vs. Low Expression Cohort)	
		HR	Log-Rank *p*	HR	Log-Rank *p*
*HUWE1*	100%	**1.87**	**0.013**	**0.63**	**0.043**
*TNRC6B*	100%	**1.73**	**0.0025**	1.31	0.11
*EEF1D*	100%	**0.70**	**0.026**	**0.73**	**0.039**
*CDK6*	74%	**1.71**	**0.013**	1.39	0.053
*CSNK1A1*	100%	1.41	0.11	**1.71**	**0.034**
*EGR1*	100%	1.36	0.055	**1.52**	**0.016**
*CDKN1A*	100%	**0.63**	**0.038**	1.49	0.076
*ITGA5*	100%	**1.51**	**0.031**	**1.35**	**0.045**
*SRRM2*	100%	**1.51**	**0.039**	1.20	0.27
*PTEN*	100%	**1.9**	**0.017**	1.34	0.12
*SMARCC1*	100%	0.84	0.43	**0.70**	**0.018**
*MGA*	99%	**1.44**	**0.023**	1.31	0.12
*VIM*	100%	**1.88**	**0.0055**	1.46	0.056
*TNRC6A*	100%	1.39	0.22	**0.73**	**0.043**
*YWHAQ*	100%	**0.62**	**0.0096**	**1.39**	**0.046**
*CLTC*	100%	**0.40**	**0.0039**	**0.59**	**0.021**
*UBE2Z*	100%	0.77	0.28	0.72	0.17
*SMAD2*	100%	**0.62**	**0.0077**	**0.60**	**0.0022**
*POLR2A*	100%	**1.49**	**0.04**	**1.67**	**0.0053**

* All genes fulfilled a cut-off of CPM ≥ 10 in ≥50% of samples from TCGA-OV and were subjected to further analyses. **CPM**—counts per million, **HR**—hazard ratio, **OS**—overall survival, **PFI**—progression-free interval. *VIM* was considered a prognostic gene due to the highest significant HR for OS (*p* = 0.0055) correlating with HR for PFI, which almost reached statistical significance (*p* = 0.056).

## Data Availability

The original TCGA data are a publicly available dataset downloaded from the National Cancer Institute Genomic Data Commons Data Portal. The data generated during the study are available from the corresponding author upon reasonable request.

## Supplementary Materials

The following supporting information can be downloaded at: https://www.mdpi.com/article/10.3390/cells13100867/s1. Supplementary Methods: Screening of endogenous control genes for qPCR studies with Custom TaqMan™ Array MicroRNA Cards; Supplementary Data: Identification of endogenous controls for use with custom TaqMan™ Array MicroRNA Cards; Figure S1: Network of miRNA–target interactions created with the MIENTURNET tool for dysregulated miRNAs identified from screening analysis with pre-designed TaqMan™ Array MicroRNA Cards and pre-selected for further validation; Figure S2: Selection of candidate endogenous control genes with the RefFinder tool based on results from pre-designed TaqMan™ Array MicroRNA Cards for further RT-qPCR studies with custom TaqMan™ Array MicroRNA Cards; Figure S3: Validation of endogenous control genes’ stable expression in HGSOC cell lines for RT-qPCR data normalization from custom TaqMan™ MicroRNA Cards; Figure S4: Results of RT-qPCR-based differential miRNA basal expression analysis in untreated PEO1, PEO1-OR, and PEO4 cell lines after 2 days of cell culture (custom TaqMan™ MicroRNA Cards); Figure S5: Results of RT-qPCR-based differential miRNA expression analysis in PEO1 cell line incubated with olaparib (O), ATRi (A), CHK1i (C), or their combinations for 2 days (custom TaqMan™ MicroRNA Cards); Figure S6: Results of RT-qPCR-based differential miRNA expression analysis in PEO1-OR cell line incubated with olaparib (O), ATRi (A), CHK1i (C), or their combinations for 2 days (custom TaqMan™ MicroRNA Cards); Figure S7: Results of RT-qPCR-based differential miRNA expression analysis in PEO4 cell line incubated with olaparib (O), ATRi (A), CHK1i (C), or their combinations for 2 days (custom TaqMan™ MicroRNA Cards); Figure S8: Network-based functional enrichment analyses of significantly differentially expressed miRNAs and their target genes in PEO1 cell line incubated with olaparib combinations for 2 days; Figure S9: Results of semi-quantitative analysis with antibody microarrays for growth factors with significantly dysregulated expression in PEO1 cells (absolute fold change ≥ 1.5, *p* < 0.05); Figure S10: Raw results of semi-quantitative analysis of 41 growth factors’ expression in PEO1 and PEO1-OR cells incubated with tested inhibitors or their combinations for 2 days; Figure S11: Kaplan–Meier plots showing the relationship between miRNAs and clinical endpoints (OS—overall survival, PFI—progression-free interval) in serous OC patients; Figure S12: Kaplan–Meier plots showing the relationship between target genes and clinical endpoints (OS—overall survival, PFI—progression-free interval) in serous OC patients; Figure S13: Kaplan–Meier plots showing the relationship between target genes and clinical endpoints (OS—overall survival, PFI—progression-free interval) in serous OC patients (continued); Table S1: List of key reagents used in the study; Table S2: Selection of 44 out of 69 dysregulated miRNAs for validation on custom TaqMan™ MicroRNA Cards based on bioinformatics analyses and literature review; Table S3: List of analyzed miRNAs and small RNAs for miRNA validation with custom TaqMan™ MicroRNA Cards; Table S4: Average fold change values of significantly differentially expressed miRNAs in HGSOC cell lines with custom TaqMan™ MicroRNA Cards; Table S5: Top significantly enriched pathways (Reactome) and biological processes (GO:BP) associated with target genes of dysregulated miRNAs in untreated PEO1-OR cells; Table S6: Top significantly enriched pathways (Reactome) associated with target genes of dysregulated miRNAs in PEO1-OR cells in response to combinations of olaparib with the ATR/CHK1 pathway inhibitors; Table S7: Experimentally validated targets of differentially expressed miRNAs from minimal subnetworks that maximally connect seeds in the PEO1-OR cell line (established with miRNet 2.0); Table S8: Identification of hub nodes using cytoHubba plug-in based on the minimal miRNA–mRNA networks using maximal clique centrality (MCC) algorithm; Table S9: Results of stage-wise differential miRNA and gene expression analysis in serous OC patients using filtered data from TCGA-OV dataset (serous OC patients with stage II, III, or IV after pharmaceutical therapy); Table S10: Validation of endogenous control genes’ stable expression in HGSOC cell lines for RT-qPCR data normalization from custom TaqMan™ MicroRNA Cards.

## Abbreviations

A	ATR inhibitor (ceralasertib)
ATR	ataxia telangiectasia and RAD3-related protein
ATRi	ATR inhibitor(s)
BRCA2	breast cancer type 2 susceptibility protein
C	CHK1 inhibitor (MK-8776)
CHK1	checkpoint kinase 1
CHK1i	CHK1 inhibitor(s)
C_T_	cycle threshold
DE	differentially expressed
DSB	double-strand break
ECACC	European Collection of Authenticated Cell Cultures
FBS	fetal bovine serum
FC	fold change
GF	growth factor
GO	Gene Ontology
HGSOC	high-grade serous ovarian cancer
HR	hazard ratio
HRD	homologous recombination deficiency
MCC	maximal clique centrality
miRNA	microRNA
O	olaparib
OC	ovarian cancer
OS	overall survival
PARP1	poly(ADP-ribose) polymerase 1
PARPi	PARP inhibitor(s)
PEO1-OR	PEO1 olaparib-resistant cell line
PFI	progression-free interval
PPI	protein–protein interaction
SD	standard deviation
TCGA	The Cancer Genome Atlas
TLDA	TaqMan^®^ Low-density Array
