# Supplementary material for: Uncovering miRNA–mRNA Regulatory Networks Related to Olaparib Resistance and Resensitization of BRCA2MUT Ovarian Cancer PEO1-OR Cells with the ATR/CHK1 Pathway Inhibitors
Source: Cells. 2024 May 17;13(10):867. doi: 10.3390/cells13100867 (PMC11119970; doi:10.3390/cells13100867)
Supplement: Supplementary file 1 [file cells-13-00867-s001.zip › cells-2949529-supplementary.pdf]

SUPPLEMENTARY INFORMATION

Uncovering miRNA-mRNA regulatory networks related to olaparib resistance and resensitization of *BRCA2*^{MUT} ovarian cancer PEO1-OR cells with the ATR/CHK1 pathway inhibitors

Łukasz Biegała[1,2], Damian Kołat[3,4], Arkadiusz Gajek[1], Elżbieta Płuciennik[3], Agnieszka Marczak[1], Agnieszka Śliwińska[5,*], Michał Mikula[6], Aneta Rogalska[1,*]

TABLE OF CONTENTS

► Supplementary Methods:

Screening of endogenous control genes for qPCR studies with Custom TaqMan™ Array MicroRNA Cards.

► Supplementary Data:

Identification of endogenous controls for use with custom TaqMan™ Array MicroRNA Cards.

► Supplementary Figures:

Figure S1: Network of miRNA-target interactions created with the MIENTURNET tool for dysregulated miRNAs identified from screening analysis with pre-designed TaqMan™ Array MicroRNA Cards and pre-selected for further validation.

Figure S2: Selection of candidate endogenous control genes with the RefFinder tool based on results from Pre-designed TaqMan™ Array MicroRNA Cards for further RT-qPCR studies with Custom TaqMan™ Array MicroRNA Cards.

Figure S3: Validation of endogenous control genes stable expression in HGSOC cell lines for RT-qPCR data normalization from Custom TaqMan™ MicroRNA Cards.

Figure S4: Results of RT-qPCR-based differential miRNA basal expression analysis in untreated PEO1, PEO1-OR, and PEO4 cell lines after 2 days of cell culture (Custom TaqMan™ MicroRNA Cards).

Figure S5: Results of RT-qPCR-based differential miRNA expression analysis in PEO1 cell line incubated with olaparib (O), ATRi (A), CHK1i (C), or their combinations for 2 days (Custom TaqMan™ MicroRNA Cards).

Figure S6: Results of RT-qPCR-based differential miRNA expression analysis in PEO1-OR cell line incubated with olaparib (O), ATRi (A), CHK1i (C), or their combinations for 2 days (Custom TaqMan™ MicroRNA Cards).

Figure S7: Results of RT-qPCR-based differential miRNA expression analysis in PEO4 cell line incubated with olaparib (O), ATRi (A), CHK1i (C), or their combinations for 2 days (Custom TaqMan™ MicroRNA Cards).

Figure S8: Network-based functional enrichment analyses of significantly differentially expressed miRNAs and their target genes in PEO1 cell line incubated with olaparib combinations for 2 days.

Figure S9: Results of semi-quantitative analysis with antibody microarrays for growth factors with significantly dysregulated expression in PEO1 cells (absolute fold change ≥ 1.5, $p < 0.05$).

Figure S10: Raw results of semi-quantitative analysis of 41 growth factors expression in PEO1 and PEO1-OR cells incubated with tested inhibitors or their combinations for 2 days.

Figure S11: Kaplan-Meier plots showing the relationship between miRNAs and clinical endpoints (OS – overall survival, PFI – progression-free intervals) in serous OC patients.

Figure S12: Kaplan-Meier plots showing the relationship between target genes and clinical endpoints (OS – overall survival, PFI – progression-free intervals) in serous OC patients.

Figure S13: Kaplan-Meier plots showing the relationship between target genes and clinical endpoints (OS – overall survival, PFI – progression-free intervals) in serous OC patients (continued).

► Supplementary Tables:

Table S1: List of key reagents used in the study.

Table S2: Selection of 44 out of 69 dysregulated miRNAs for validation on Custom TaqMan™ MicroRNA Cards based on bioinformatics analyses and literature review.

Table S3: List of analyzed miRNAs and small RNAs for miRNA validation with Custom TaqMan™ MicroRNA Cards.

Table S4: Average fold change values of significantly differentially expressed miRNAs in HGSOC cell lines with (Custom TaqMan™ MicroRNA Cards).

Table S5: Top significantly enriched pathways (Reactome) and biological processes (GO:BP) associated with target genes of dysregulated miRNAs in untreated PEO1-OR cells.

Table S6: Top significantly enriched pathways (Reactome) associated with target genes of dysregulated miRNAs in PEO1-OR cells in response to combinations of olaparib with the ATR/CHK1 pathway inhibitors.

Table S7: Experimentally validated targets of differentially expressed miRNAs from minimal subnetworks that maximally connect seeds in the PEO1-OR cell line (established with miRNet 2.0).

Table S8: Identification of hub nodes using CytoHubba plug-in based on the minimal miRNA-mRNA networks using maximal clique centrality (MCC) algorithm.

Table S9: Results of stage-wise differential miRNA and gene expression analysis in serous OC patients using filtered data from TCGA-OV dataset (serous OC patients with stage II, III, or IV after pharmaceutical therapy).

Table S10: Validation of endogenous control genes stable expression in HGSOC cell lines for RT-qPCR data normalization from Custom TaqMan™ MicroRNA Cards.

SUPPLEMENTARY METHODS

Screening and validation of endogenous control genes for qPCR studies with Custom TaqMan™ Array MicroRNA Cards

The global mean normalization method, used to normalize data from RT-qPCR miRNA profiling studies with Pre-designed TaqMan™ Array MicroRNA Cards, is accurate in studies where many miRNAs are tested per sample. Custom TaqMan™ Array MicroRNA Cards contain one assay for U6 snRNA as a common candidate endogenous control gene by default. To identify additional stably expressed genes for data normalization before the design of Custom TaqMan™ Array MicroRNA Cards for 44 target miRNAs, we screened for two more reference genes based on the results from global miRNA profiling studies for 754 miRNAs. Firstly, raw C_T data from screening experiments was filtered to consider miRNAs which were reliably detected in all untreated and treated samples in PEO1 and PEO1-OR cells (C_T value < 35) and were abundantly expressed in both cell lines (average C_T value < 28). Briefly, suitable reference genes were identified based on gene stability rankings calculated with the RefFinder web-based tool. This algorithm allows the computation of the overall final ranking based on weights calculated with four different programs (geNorm, NormFinder, BestKeeper, and the comparative ΔC_T method). Genes were prioritized according to the highest geometric mean of weights for the final rankings (stability), lowest C_T value (expression level), and lowest standard deviations of C_T (variability among groups). One gene for small RNA (RNU48 snoRNA) and one for miRNA (miRNA-30e-3p) with the highest expression stability, lowest standard deviation among groups, and highest expression level (lowest C_T) were selected as additional candidates for reference genes in further quantitative RT-qPCR analyses.

SUPPLEMENTARY DATA

Identification of endogenous controls for use with custom TaqMan™ Array MicroRNA Cards

Small-scale miRNA gene expression studies should be preceded by a selection of the most stable small RNA controls for accurate normalization of miRNA expression data. Traditional use of a single gene for normalization might lead to relatively substantial errors. Hence, we used prior RT-qPCR data from large-scale miRNA profiling with predesigned TaqMan™ Array MicroRNA Cards to identify three reliable endogenous controls for use in normalization by multiple housekeeping genes with Custom Cards. We initially confirmed the stability of candidate endogenous control introduced on cards by default (U6 snRNA) and recommended by the manufacturer (RNU48) in both PEO1 and PEO1-OR cells. Comparison of a geometric mean of ranking values from RefFinder, average C_T values, and standard deviation of C_T for genes included in the analysis are presented in Figure S2. Out of all included miRNAs, we have evaluated 11 miRNAs meeting the inclusion criteria. miR-30e-3p was selected as the most stably and relatively abundantly expressed miRNA upon tested treatment conditions based on summed gene stability ranking evaluated with RefFinder tool, calculated average C_T values and its average standard deviation ($C_T = 26.7 \pm 0.17$) in both PEO1 and PEO1-OR cells (Figure S2).

Three pre-selected endogenous control genes (U6 snRNA, RNU48 snoRNA, and miR-30e-3p) were evaluated for stable expression across treatment conditions in PEO1, PEO4, and PEO1-OR cell lines to confirm their stability in the final qPCR-based miRNA quantification experiments with Custom TaqMan™ Array MicroRNA Cards. The average C_T values and standard deviation of C_T and average C_T range across treatments in all HGSOC cell lines (Table S10) for all three endogenous control genes were used as a direct measure of the stability of these genes.

The low variation of expression values (C_T range of 0.19 – 0.67 and SD of average C_T of 0.08 – 0.22) indicated a very small dispersion among samples under studied treatment conditions (Table 2). C_T values for each group were presented on graphs in Figure S3. Comparison of C_T means among untreated and treated groups in all HGSOC cell lines showed no statistically significant difference for endogenous control gene expression (Table S10 and Figure S3). Hence, a multiple reference gene normalization strategy was applied to normalize RT-qPCR miRNA expression data for 44 target miRNAs.

SUPPLEMENTARY FIGURES

Supplementary Figure 1 (Figure S1)

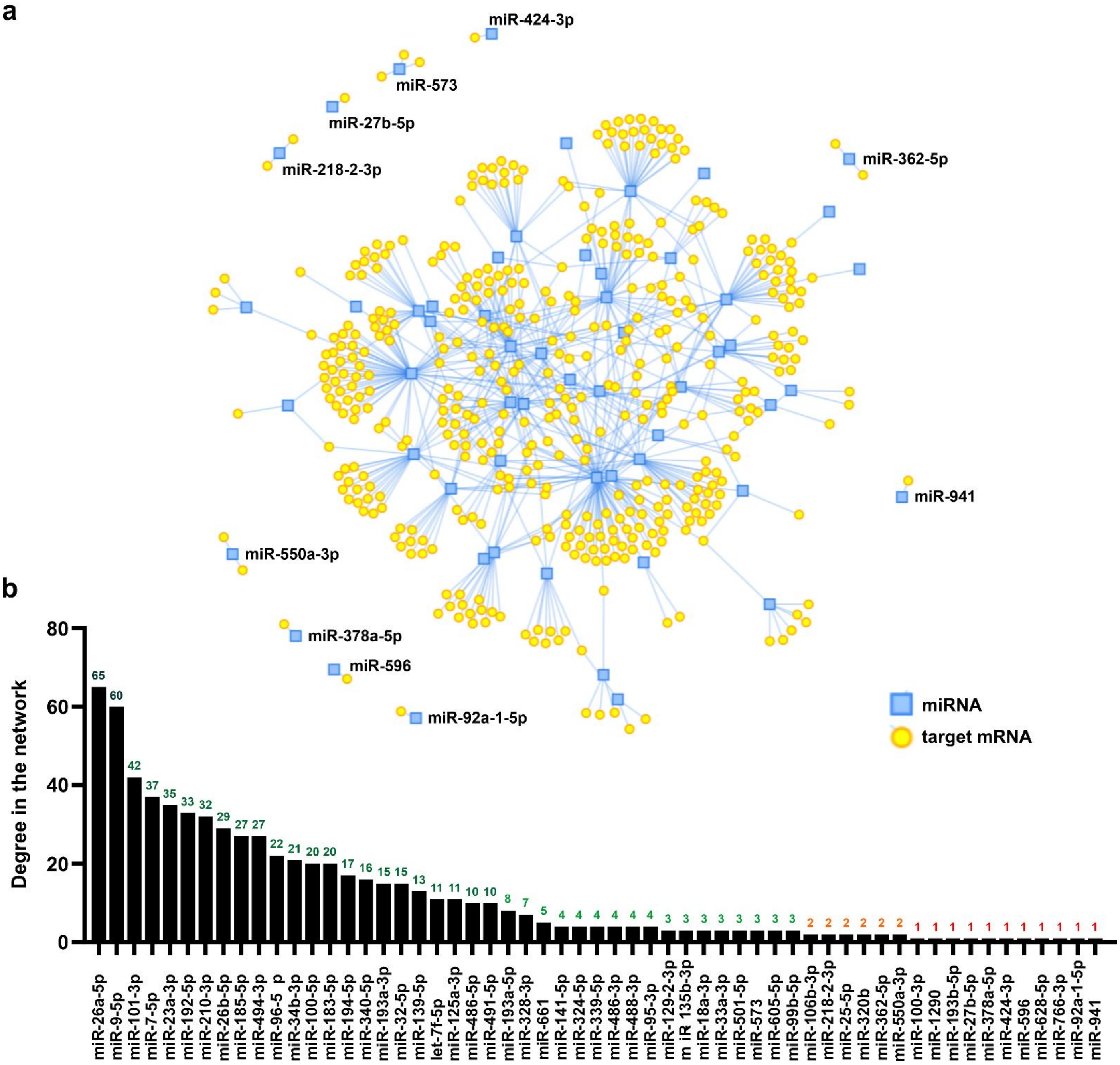

Figure S1. Network of miRNA-target interactions created with the MIENTURNET tool for dysregulated miRNAs identified from screening analysis with pre-designed TaqMan™ Array MicroRNA Cards and pre-selected for further validation. (a) Visualization of miRNA-target interaction network. Blue squares refer to miRNAs, while yellow circles refer to their target genes. miRNA not connected with the network were highlighted by their names. **(b)** Bar plot representing network degree for miRNAs.

Supplementary Figure 2 (Figure S2)

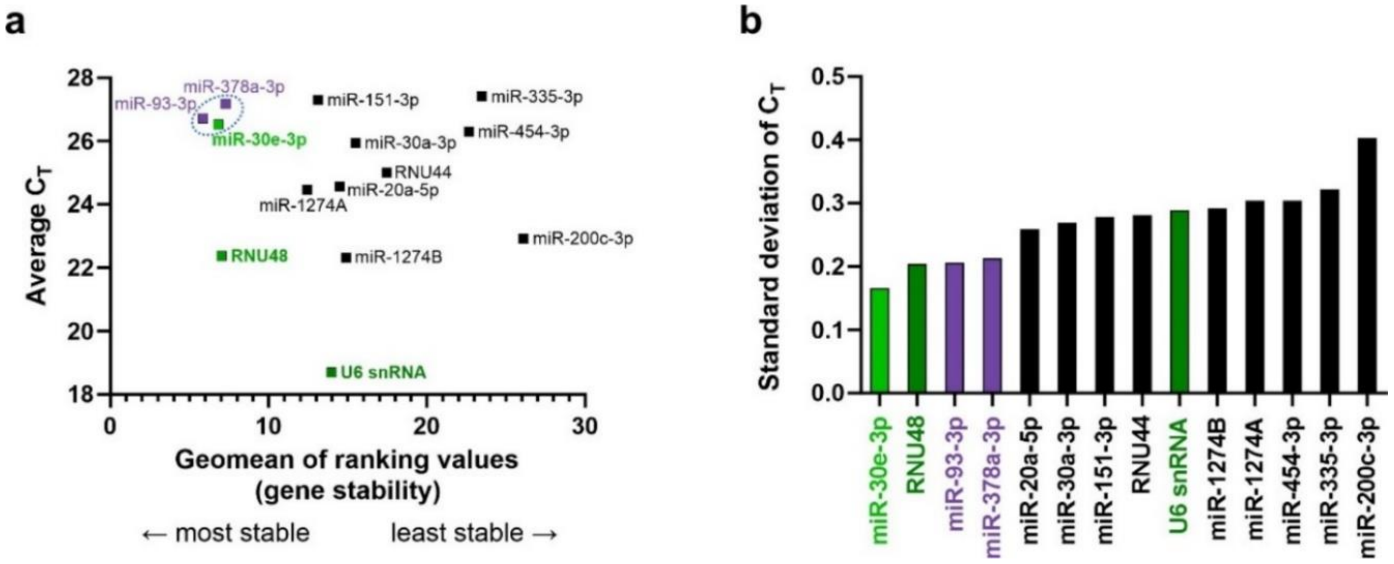

Figure S2. Selection of candidate endogenous control genes with the RefFinder tool based on results from Pre-designed TaqMan™ Array MicroRNA Cards for further RT-qPCR studies with Custom TaqMan™ Array MicroRNA Cards. Raw C_T values for each MicroRNA or gene were used to identify the most stable reference genes among groups in untreated and treated PEO1 and PEO1-OR cells. One small RNA from each class (snRNA, snoRNA, and miRNA) was selected as a reference gene. U6 snRNA is a fixed endogenous control introduced on TLDA Cards by the manufacturer. **(a)** Evaluation of candidate gene expression stability based on their geometric mean of ranking values calculated with RefFinder (x-axis) and average C_T values (expression level on y-axis). Undetected miRNAs in any group ($C_T \geq 35$) and detected miRNAs with low expression among groups ($C_T \geq 28$) were excluded from the analysis. The remaining miRNAs were used to evaluate their stability using the RefFinder web-based tool. Reference genes were prioritized according to their stability rankings derived from the four programs (geNorm, NormFinder, BestKeeper, and the comparative ΔC_T method). Geomean of ranking values is a sum of values calculated separately for PEO1 and PEO1-OR cells. Averaged C_T is a mean value calculated separately for PEO1 and PEO1-OR cells. RNU48 exhibited the highest stability among the two analyzed snRNAs and was selected as the second reference gene. Among all analyzed miRNAs, three showed the highest gene stability and similarly modern expression levels (C_T from 26.7 – 27.2): miR-30e-3p, miR93-3p, and miR-378a-3p (marked with a blue oval). **(b)** The average standard deviation of C_T values for miRNAs among groups was used to select miRNA with the lowest expression variability. Out of three miRNAs pre-selected with RefFinder (miR-30e-3p, miR93-3p, and miR-378a-3p), miR-30e-3p expression was the most consistent among groups (SD = 0.17). Moreover, it showed the lowest average C_T and it was selected as the third reference gene.

Supplementary Figure 3 (Figure S3)

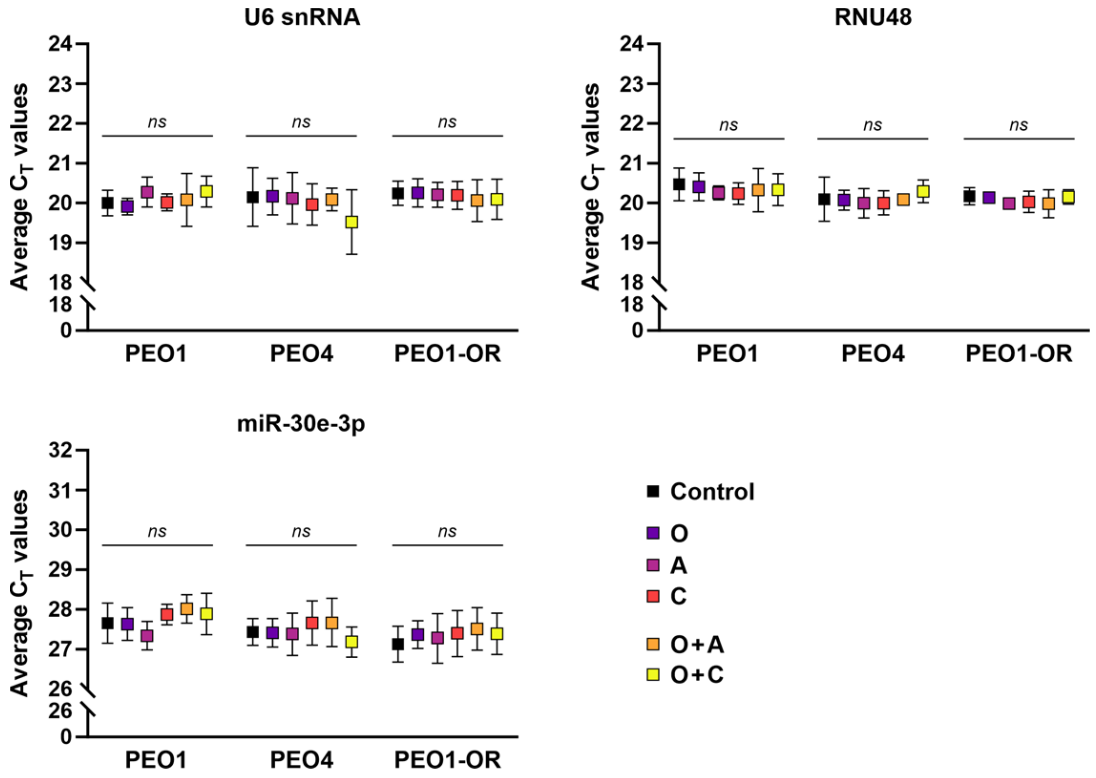

Figure S3. Validation of endogenous control genes stable expression in HGSOC cell lines for RT-qPCR data normalization from Custom TaqMan™ MicroRNA Cards. Symbols on graphs represent average C_T values (± SD) from four independent biological replicates (n = 4). Significant differences in average C_T values between treatments for each gene and cell line were evaluated with ordinary one-way ANOVA followed by Tukey's post-hoc test. The abbreviation "*ns*" indicates a nonsignificant difference. O – olaparib, A – ATRi, C – CHK1i.

Supplementary Figure 4 (Figure S4)

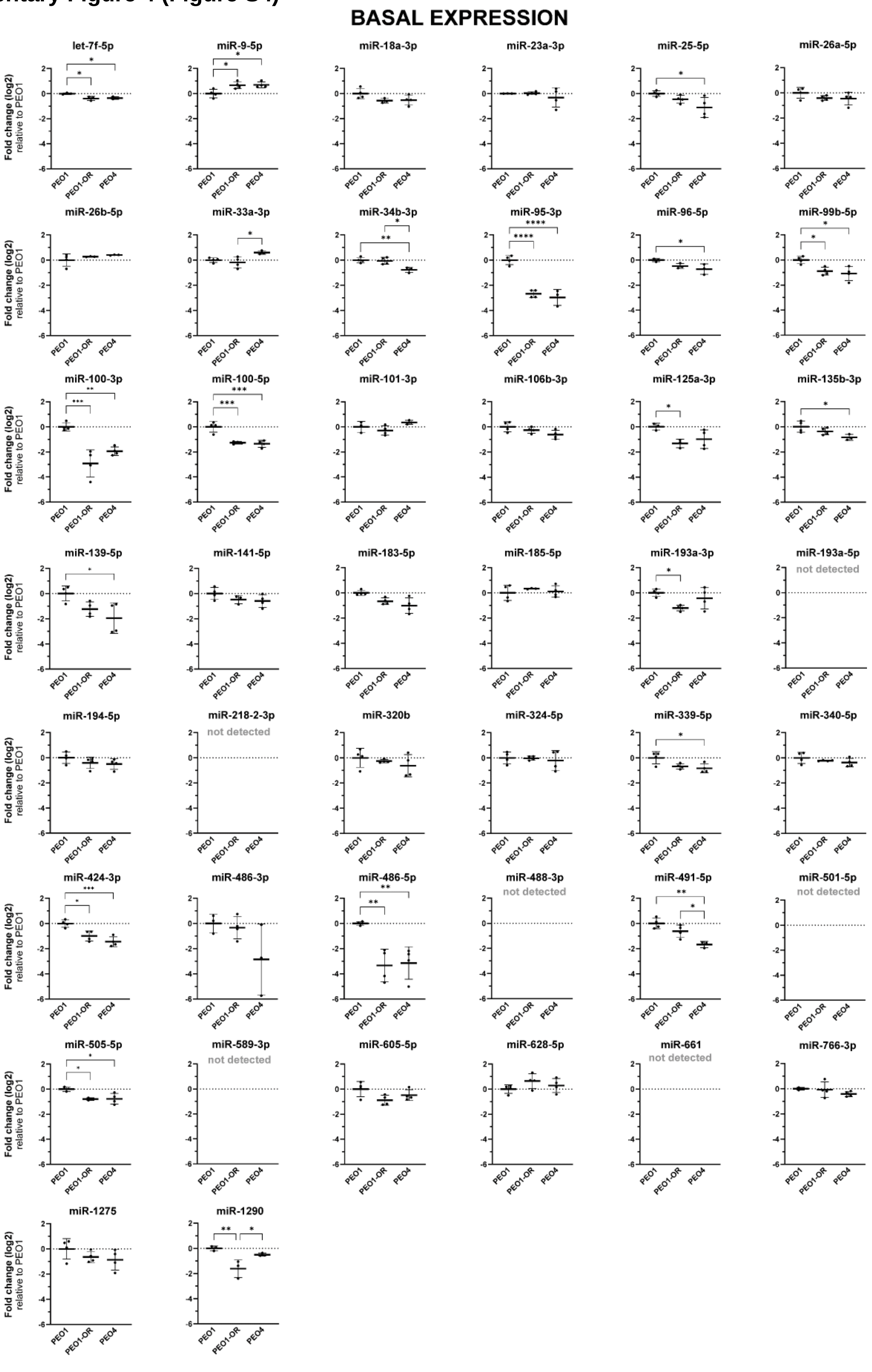

Figure S4. Results of RT-qPCR-based differential miRNA basal expression analysis in untreated PEO1, PEO1-OR, and PEO4 cell lines after 2 days of cell culture (Custom TaqMan™ MicroRNA Cards). Relative levels of miRNAs were expressed as means of logarithmic fold change ± SD (n = 3 – 4). Statistical significance was assessed compared to PEO1 cells with one-way ANOVA followed by multiple comparison tests: *$p < 0.05$, **$p < 0.01$, ***$p < 0.001$, ****$p < 0.0001$.

Supplementary Figure 5 (Figure S5)

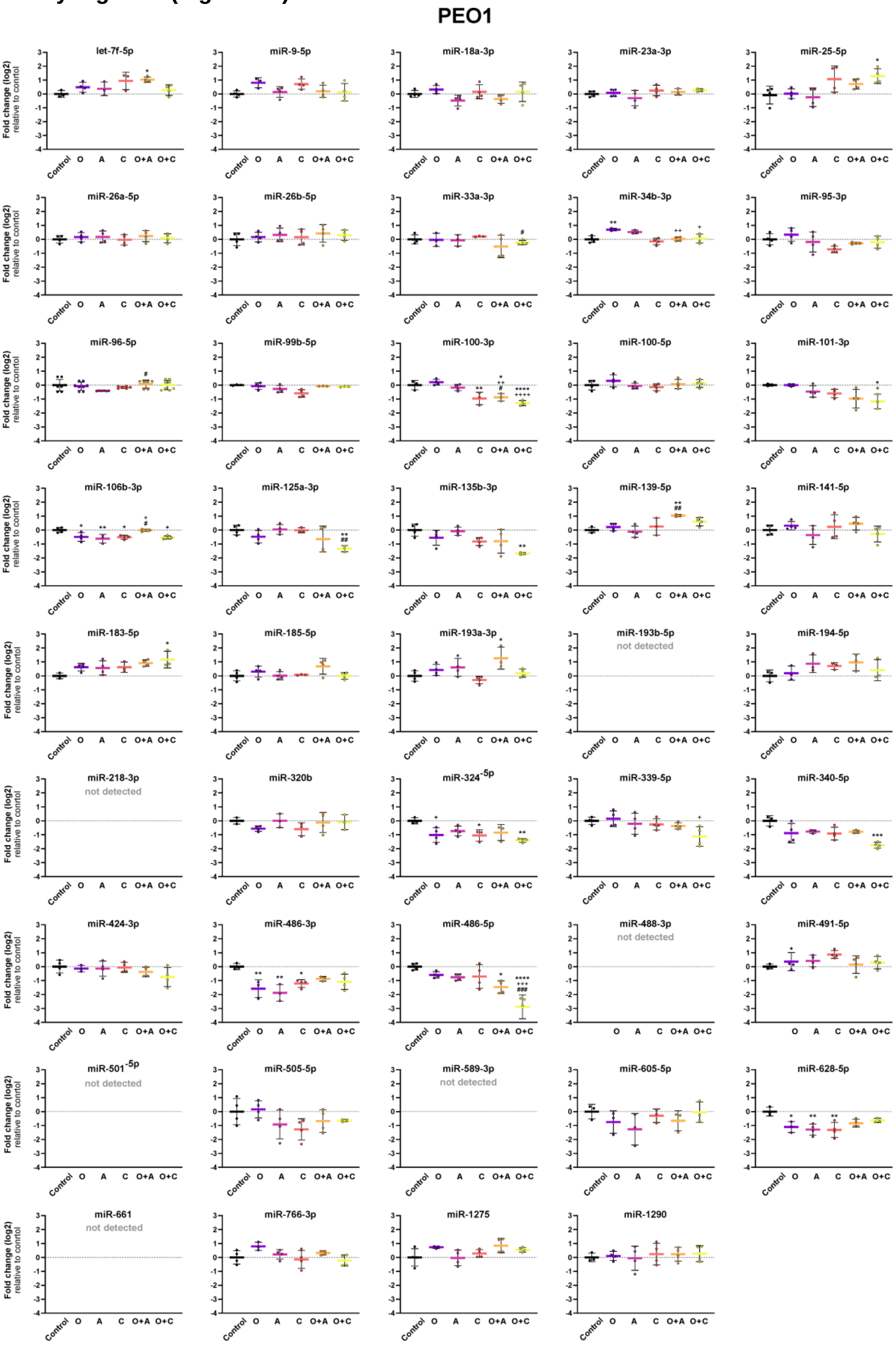

Figure S5. Results of RT-qPCR-based differential miRNA expression analysis in PEO1 cell line incubated with olaparib (O), ATRi (A), CHK1i (C), or their combinations for 2 days (Custom TaqMan™ MicroRNA Cards). Relative levels of miRNAs were expressed as means of logarithmic fold change ± SD (n = 3 – 4). Statistical significance was assessed with one-way ANOVA followed by multiple comparison tests: $^{*}p < 0.05$, $^{**}p < 0.01$, $^{***}p < 0.001$, $^{****}p < 0.0001$ (treatment vs. control); $^{+}p < 0.05$, $^{++}p < 0.01$, $^{+++}p < 0.001$, $^{++++}p < 0.0001$ (O vs. combination with A or C); $^{\#}p < 0.05$, $^{\#\#}p < 0.01$, $^{\#\#\#}p < 0.001$, $^{\#\#\#\#}p < 0.0001$ (A or C vs. respective combinations with O).

Supplementary Figure 6 (Figure S6)

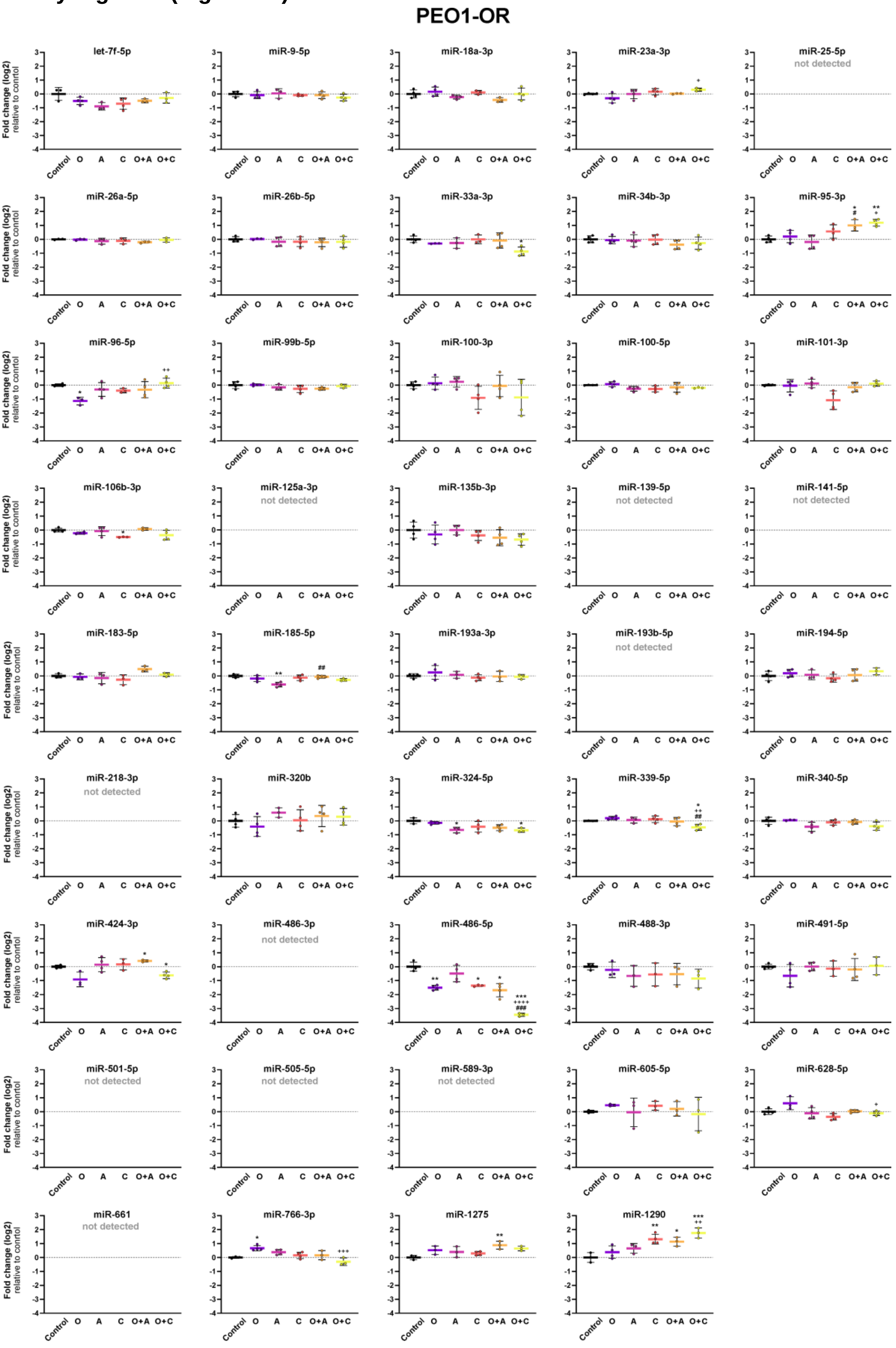

Figure S6. Results of RT-qPCR-based differential miRNA expression analysis in PEO1-OR cell line incubated with olaparib (O), ATRi (A), CHK1i (C), or their combinations for 2 days (Custom TaqMan™ MicroRNA Cards). Relative levels of miRNAs were expressed as means of logarithmic fold change ± SD (n = 3 – 4). Statistical significance was assessed with one-way ANOVA followed by multiple comparison tests: $^{*}p < 0.05$, $^{**}p < 0.01$, $^{***}p < 0.001$, $^{****}p < 0.0001$ (treatment vs. control); $^{+}p < 0.05$, $^{++}p < 0.01$, $^{+++}p < 0.001$, $^{++++}p < 0.0001$ (O vs. combination with A or C); $^{\#}p < 0.05$, $^{\#\#}p < 0.01$, $^{\#\#\#}p < 0.001$, $^{\#\#\#\#}p < 0.0001$ (A or C vs. respective combinations with O).

Supplementary Figure 7 (Figure S7)

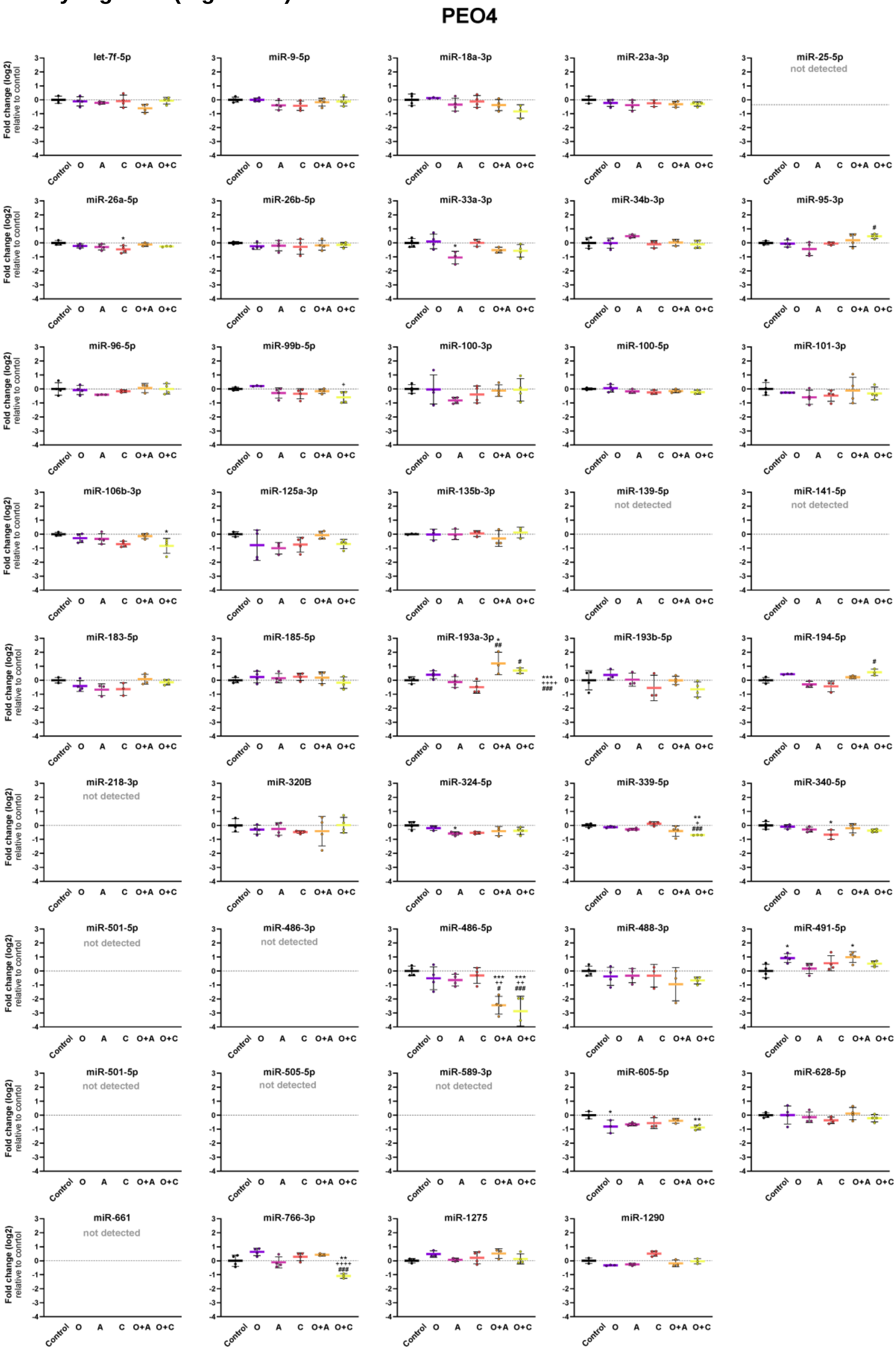

Figure S7. Results of RT-qPCR-based differential miRNA expression analysis in PEO4 cell line incubated with olaparib (O), ATRi (A), CHK1i (C), or their combinations for 2 days (Custom TaqMan™ MicroRNA Cards). Relative levels of miRNAs were expressed as means of logarithmic fold change ± SD (n = 3 – 4). Statistical significance was assessed with one-way ANOVA followed by multiple comparison tests: $^{*}p < 0.05$, $^{**}p < 0.01$, $^{***}p < 0.001$, $^{****}p < 0.0001$ (treatment vs. control); $^{+}p < 0.05$, $^{++}p < 0.01$, $^{+++}p < 0.001$, $^{++++}p < 0.0001$ (O vs. combination with A or C); $^{\#}p < 0.05$, $^{\#\#}p < 0.01$, $^{\#\#\#}p < 0.001$, $^{\#\#\#\#}p < 0.0001$ (A or C vs. respective combinations with O).

Supplementary Figure 8 (Figure S8)

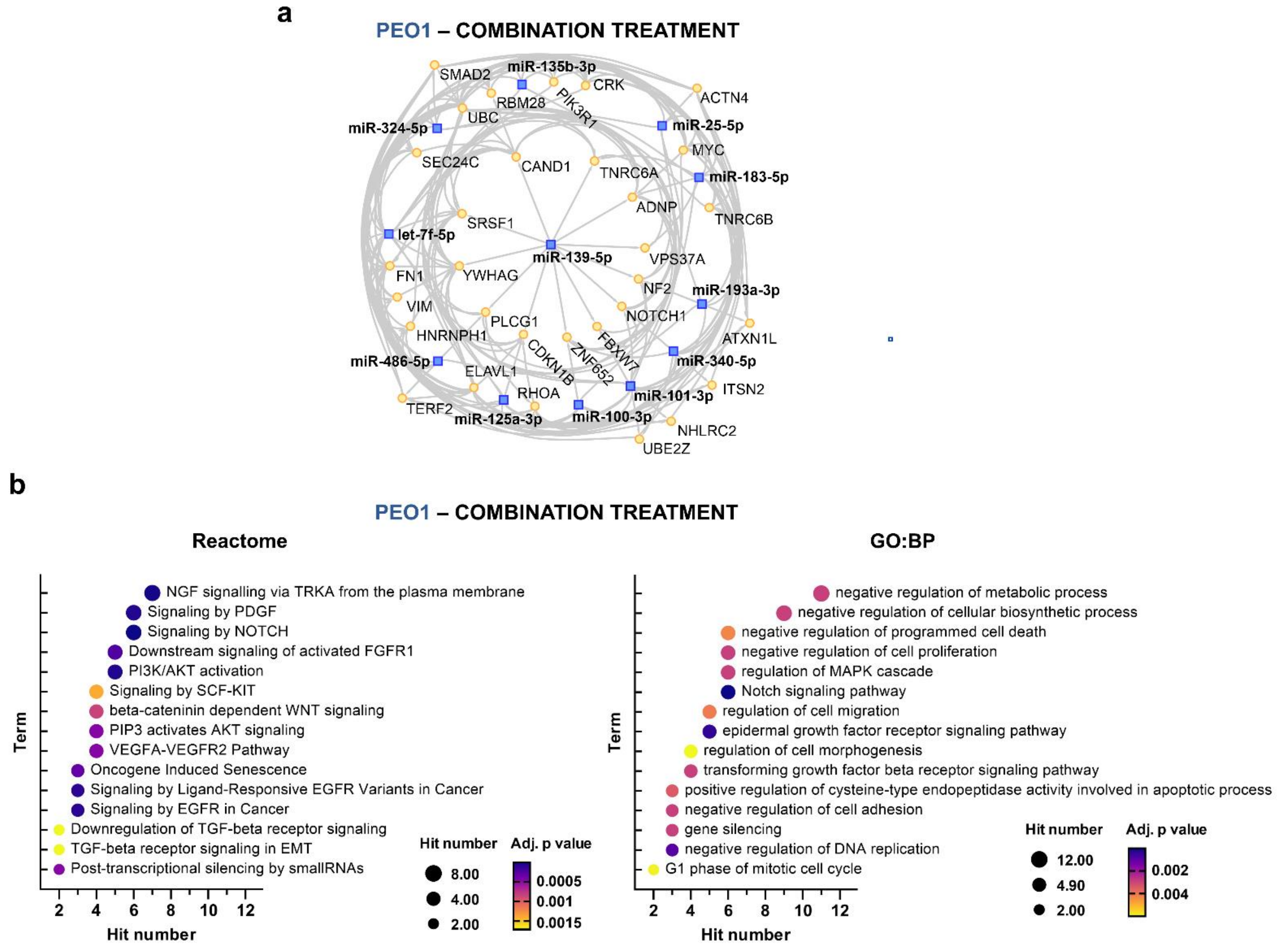

Figure S8. Network-based functional enrichment analyses of significantly differentially expressed miRNAs and their target genes in PEO1 cell line incubated with olaparib combinations for 2 days. (a) The minimal miRNA-mRNA interaction network. The blue square nodes represent miRNAs, and the yellow circular nodes represent target genes. **(b)** Enrichment terms visualized with bubble plots based on over-representation analysis for differentially expressed miRNA target genes in PEO1 cells. The most significantly enriched functional annotations were selected following analysis with Reactome pathways and Gene Ontology biological process (GO:BP) databases. Terms were ranked by adjusted *p* value and number of target genes (hit).

Supplementary Figure 9 (Figure S9)

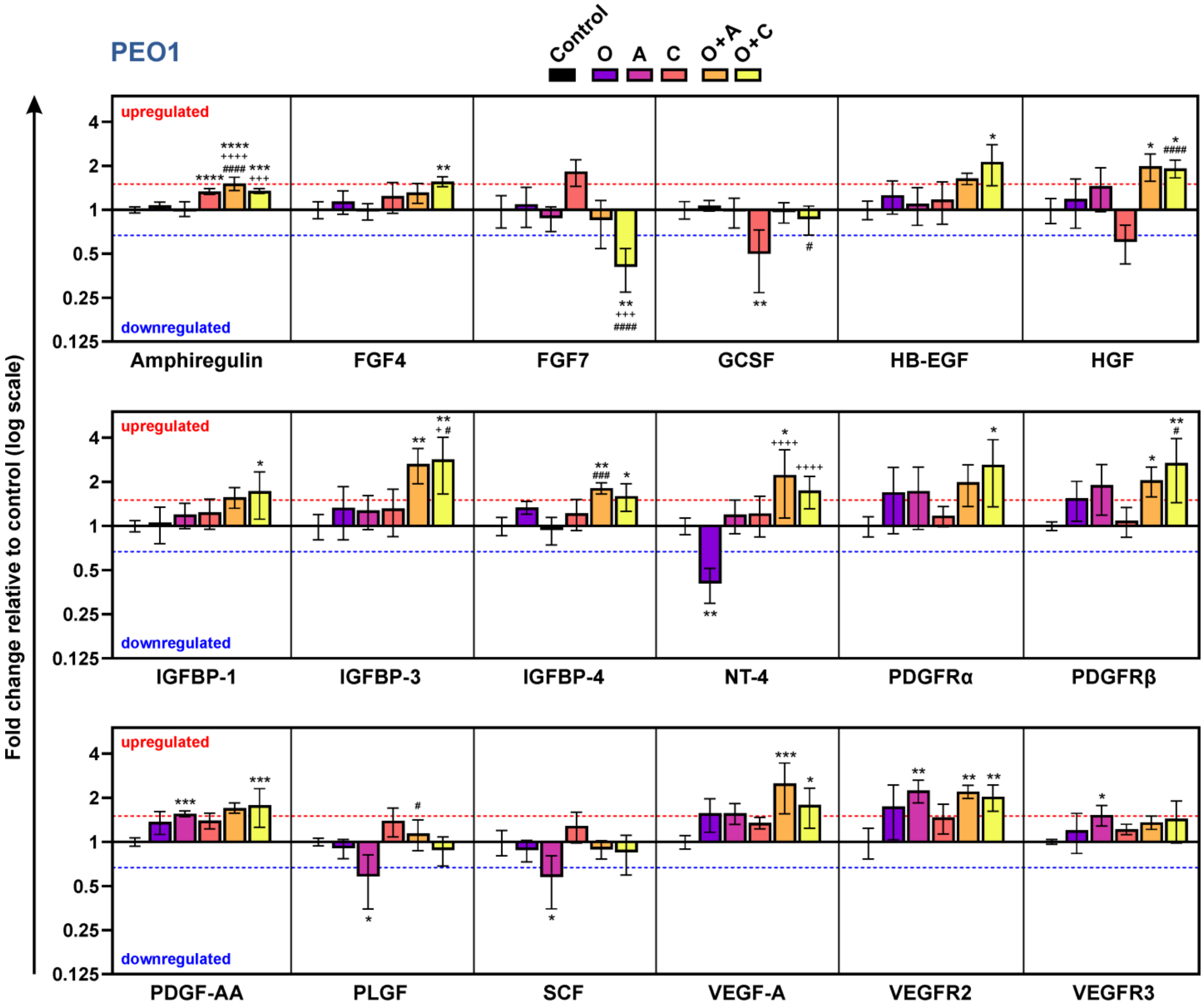

Figure S9. Results of semi-quantitative analysis with antibody microarrays for growth factors with significantly dysregulated expression in PEO1 cells (absolute fold change ≥ 1.5, *p* < 0.05). Cells were incubated with inhibitors (O, A, C) or their combinations (O + A, O + C) for 2 days. Data was expressed as mean fold change ± SD (n = 4) on a logarithmized scale relative to untreated control cells. Statistical significance was assessed using one-way ANOVA followed by multiple comparison tests: $^{*}p < 0.05$, $^{**}p < 0.01$, $^{***}p < 0.001$, $^{****}p < 0.0001$ (treatment vs. control); $^{+}p < 0.05$, $^{++}p < 0.01$ (O vs. combination with A or C); $^{\#}p < 0.05$, $^{\#\#}p < 0.01$, $^{\#\#\#}p < 0.001$, $^{\#\#\#\#}p < 0.0001$ (A or C vs. respective combinations with O). O – olaparib, A – ATRi, C – CHK1i.

Supplementary Figure 10 (Figure S10)

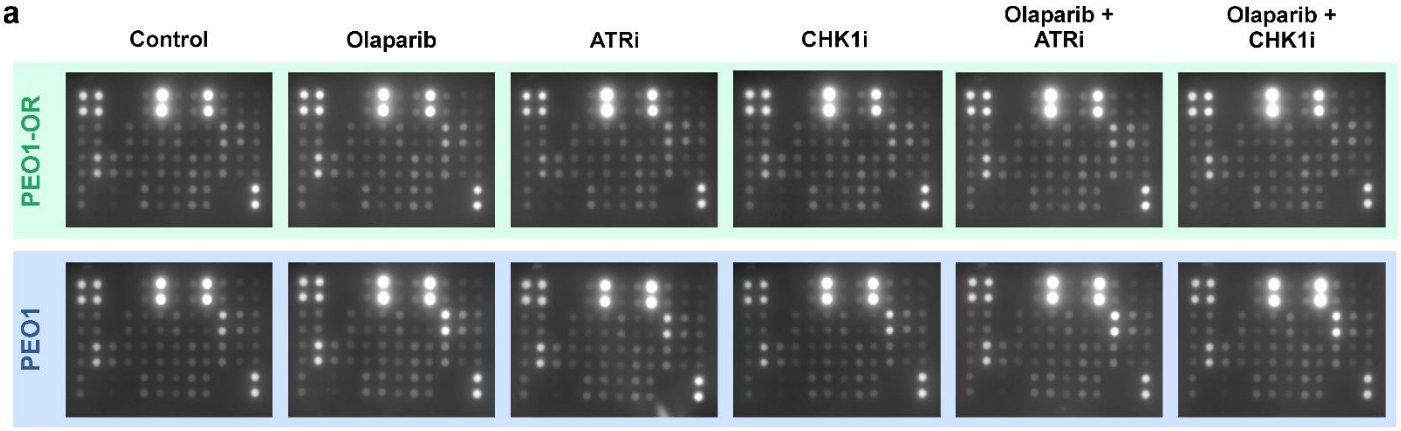

b

	A	B	C	D	E	F	G	H	I	J	K	L
1–2	POS	POS	NEG	NEG	Amphiregulin	bFGF	βNGF	EGF	EGFR	FGF-4	FGF-6	FGF-7
3–4	GCSF	GDNF	GM-CSF	HB-EGF	HGF	IGFBP-1	IGFBP-2	IGFBP-3	IGFBP-4	IGFBP-6	IGF-1	IGF-1R
5–6	IGF-2	M-CSF	M-CSF R	NT-3	NT-4	PDGFRα	PDGFRβ	PDGF-AA	PDGF-AB	PDGF-BB	PLGF	SCF
7–8	SCFR	TGFα	TGFβ1	TGFβ2	TGFβ3	VEGF-A	VEGFR2	VEGFR3	VEGF-D	BLANK	BLANK	POS

Figure S10. Raw results of semi-quantitative analysis of 41 growth factors expression in PEO1 and PEO1-OR cells incubated with tested inhibitors or their combinations for 2 days. (a) Representative enhanced chemiluminescence images of growth factor antibody arrays for PEO1 and PEO1-OR cell lines. **(b)** Array map with the locations of individual antigen-specific antibodies spotted in duplicate vertically. POS – positive control spots used for data normalization between arrays, NEG – negative control spots used to measure the baseline signal.

Supplementary Figure 11 (Figure S11)

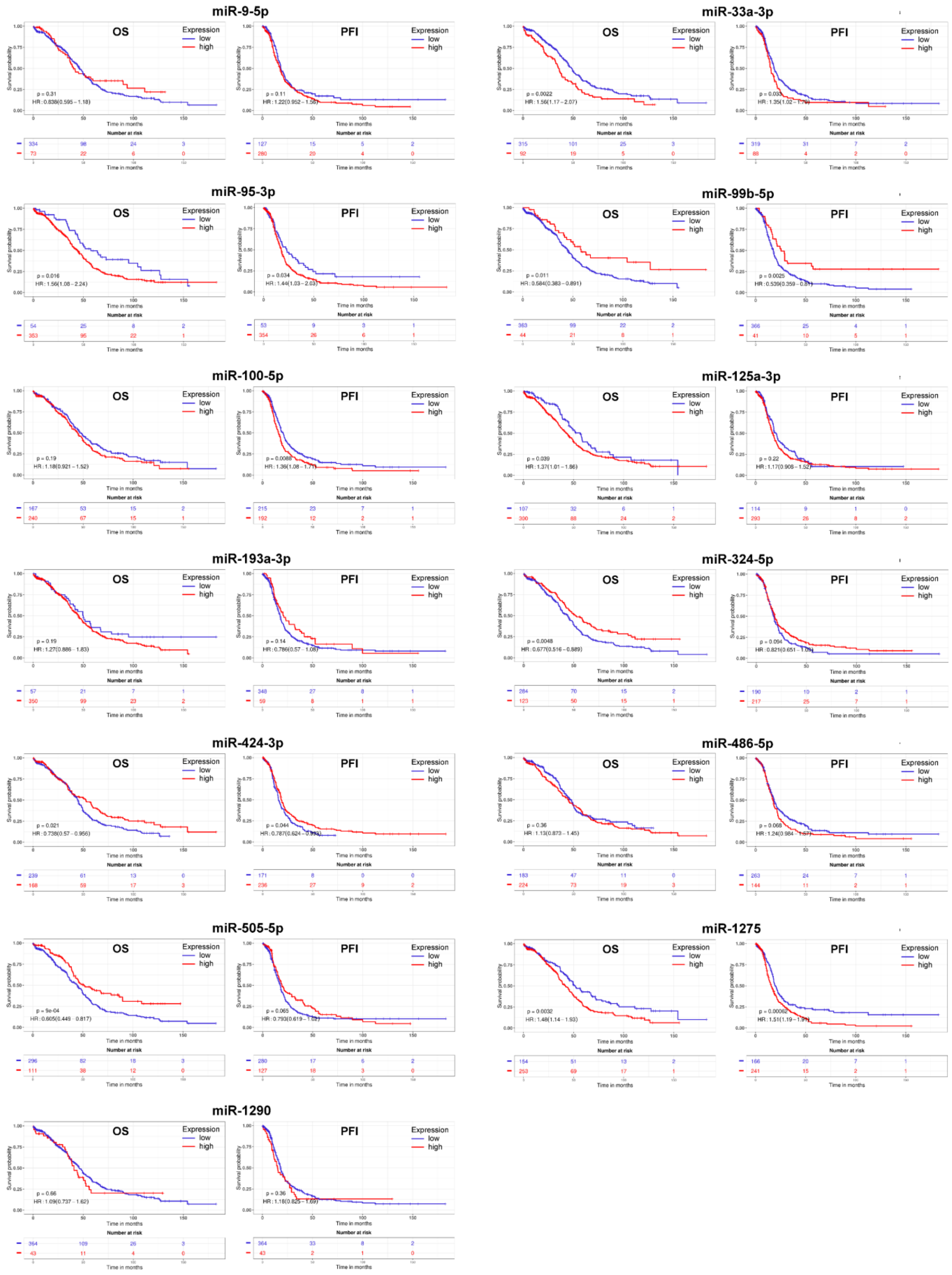

Figure S11. Kaplan-Meier plots showing the relationship between miRNAs and clinical endpoints (OS – overall survival, PFI – progression-free intervals) in serous OC patients. Prognostic univariate analyses were performed with the ToPP web-based tool with integrated data from TCGA-OV for HGSOC patients. Low and high expression OC cohorts were defined using the best cutoff and log-rank test. HR – hazard ratio (high vs low expression cohort).

Supplementary Figure 12 (Figure S12)

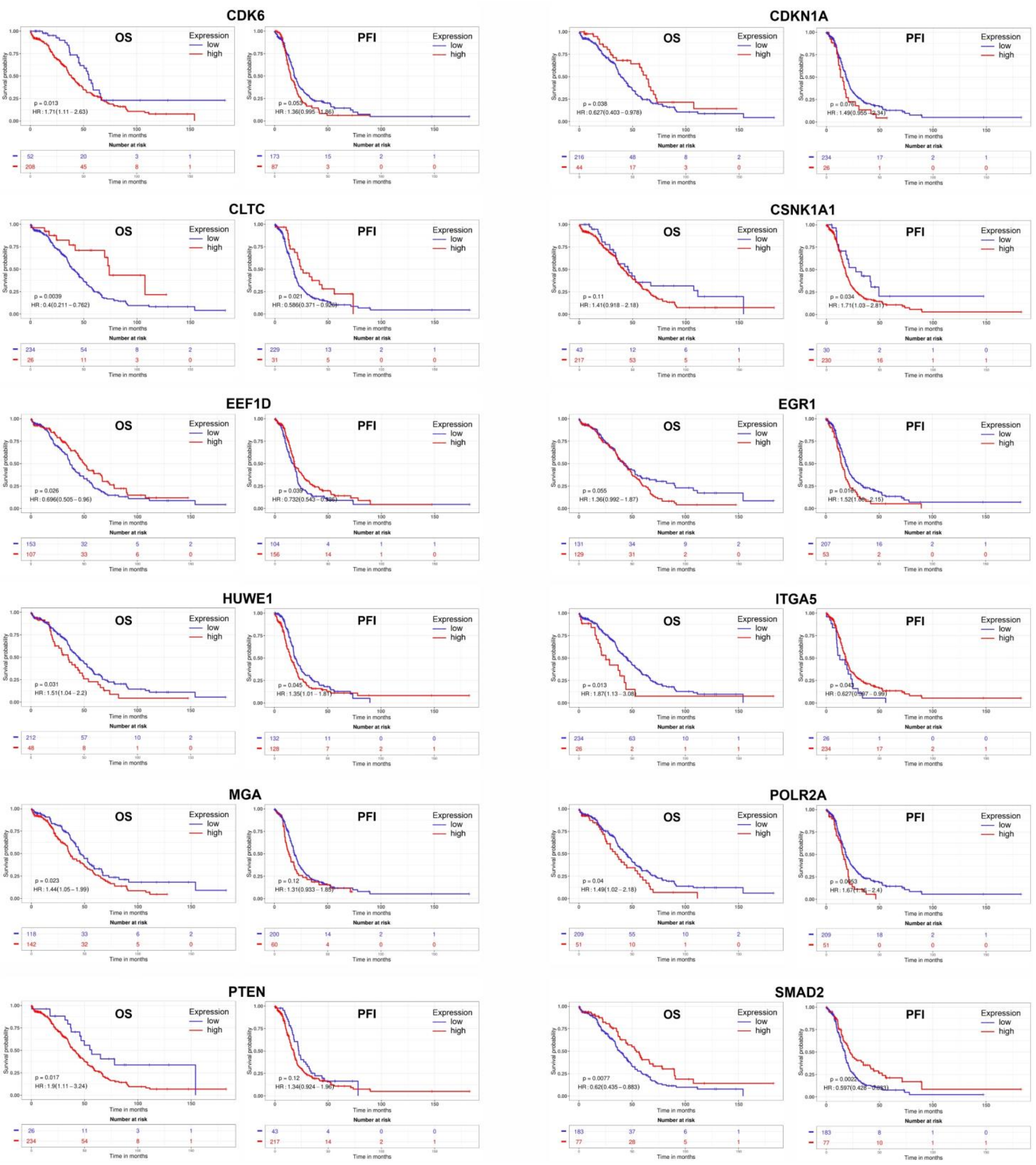

Figure S12. Kaplan-Meier plots showing the relationship between target genes and clinical endpoints (OS – overall survival, PFI – progression-free intervals) in serous OC patients. Prognostic univariate analyses were performed with the ToPP web-based tool with integrated data from TCGA-OV for HGSOC patients. Low and high expression OC cohorts were defined using the best cutoff and log-rank test. HR – hazard ratio (high vs low expression cohort).

Supplementary Figure 13 (Figure S13)

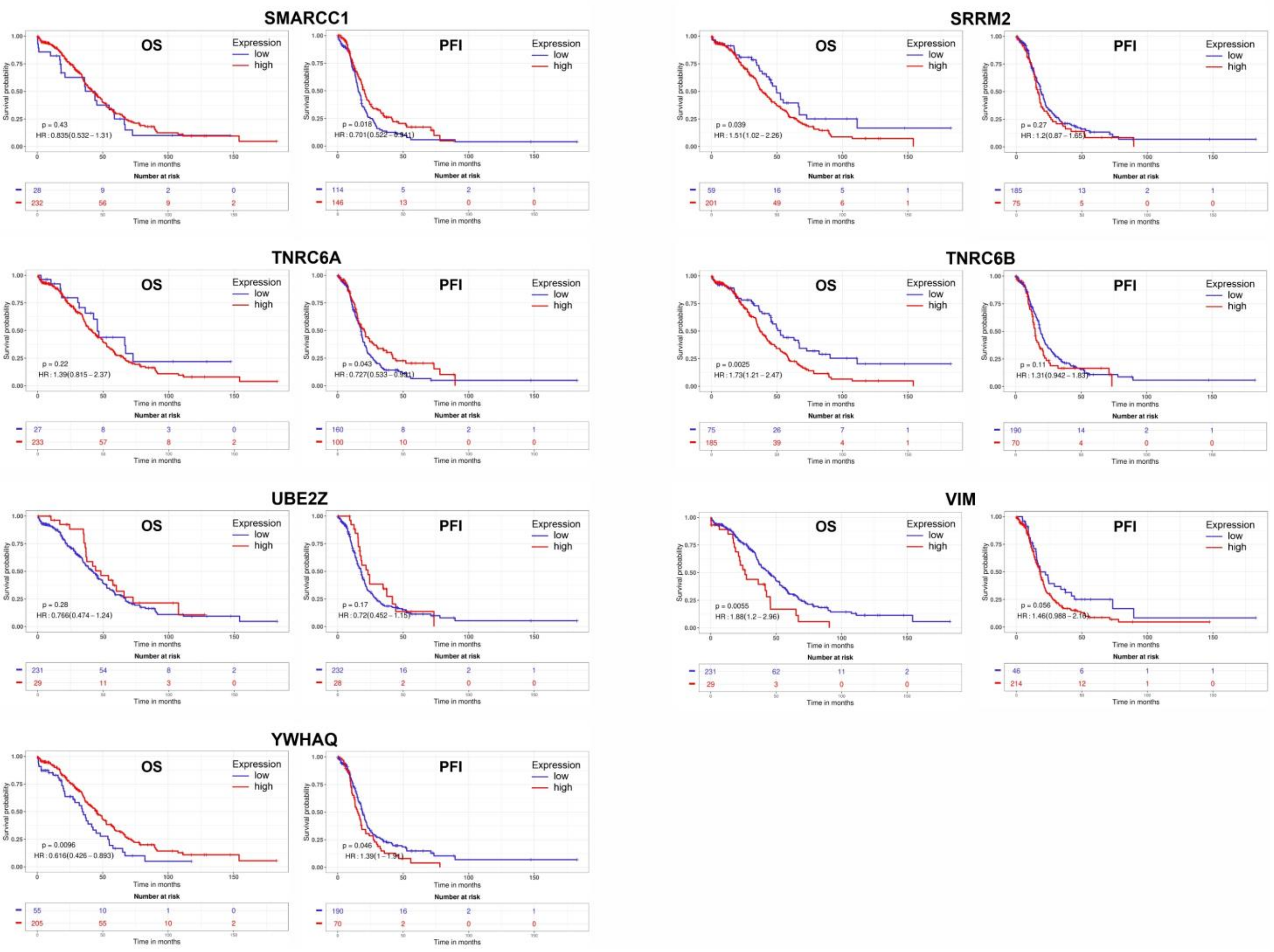

Figure S13. Kaplan-Meier plots showing the relationship between target genes and clinical endpoints (OS – overall survival, PFI – progression-free intervals) in serous OC patients (continued).

SUPPLEMENTARY TABLES

Supplementary Table 1 (Table S1)

List of key reagents used in the study.

Reagent	Catalog Number	Manufacturer
Megaplex™ RT Primers, Human Pool Set v3.0	4444745	Applied Biosystems™ (Thermo Fisher Scientific)
Custom TaqMan™ Array MicroRNA Card	4449139	Applied Biosystems™ (Thermo Fisher Scientific)
*mir*Vana™ miRNA Isolation Kit, with phenol	AM1560	Applied Biosystems™ (Thermo Fisher Scientific)
TaqMan™ MicroRNA Reverse Transcription Kit	4366596	Applied Biosystems™ (Thermo Fisher Scientific)
TaqMan™ Universal Master Mix II, no UNG	4440049	Applied Biosystems™ (Thermo Fisher Scientific)
Water, Nuclease-free, Molecular Biology Grade, Ultrapure, Thermo Scientific Chemicals	J71786.XCR	Applied Biosystems™ (Thermo Fisher Scientific)
TaqMan™ Array Human MicroRNA A+B Cards Set v3.0	4444913	Applied Biosystems™ (Thermo Fisher Scientific)
High-Capacity cDNA Reverse Transcription Kit	4368814	Applied Biosystems™ (Thermo Fisher Scientific)
RNase Inhibitor	N8080119	Applied Biosystems™ (Thermo Fisher Scientific)
Ceralasertib (ATRi)	TBW02661	Wuhan ChemNorm Biotech
MK-8776 (CHK1i)	TBW02666	Wuhan ChemNorm Biotech
Olaparib	S1060	Selleck Chemicals
RayBio® C-Series Human Growth Factor Antibody Array 1	AAH-GF-1-8	RayBiotech Life

Supplementary Table 2 (Table S2)

Selection of 44 out of 69 dysregulated miRNAs for validation on Custom TaqMan™ MicroRNA Cards based on bioinformatic analyses and literature review.

#	miRNA	miRBase accession	Strong experimental evidence of miRNA-mRNA interactions *	Interaction with the network **	Node degree in the network ≥ 3	Selected for validation
1	let-7f-5p	+	+	+	+	YES
2	let-7i-3p	+	NO	n/a	n/a	NO
3	miR-7-5p	+	+	+	+	NO
4	miR-9-5p	+	+	+	+	YES
5	miR-18a-3p	+	+	+	+	YES
6	miR-23a-3p	+	+	+	+	YES
7	miR-25-5p	+	+	+	NO	YES***
8	miR-26a-5p	+	+	+	+	YES
9	miR-26b-5p	+	+	+	+	YES
10	miR-27b-5p	+	+	NO	NO	NO
11	miR-32-5p	+	+	+	+	NO
12	miR-33a-3p	+	+	+	+	YES
13	miR-34b-3p	+	+	+	+	YES
14	miR-92a-1-5p	+	+	NO	NO	NO
15	miR-95-3p	+	+	+	+	YES
16	miR-96-5p	+	+	+	+	YES
17	miR-99b-5p	+	+	+	+	YES
18	miR-100-3p	+	+	+	NO	YES***
19	miR-100-5p	+	+	+	+	YES
20	miR-101-3p	+	+	+	+	YES
21	miR-106b-3p	+	+	+	NO	YES***
22	miR-125a-3p	+	+	+	+	YES
23	miR-129-2-3p	+	+	+	+	NO
24	miR-130b-5p	+	NO	n/a	n/a	NO
25	miR-135b-3p	+	+	+	+	NO
26	miR-139-5p	+	+	+	+	YES
27	miR-141-5p	+	+	+	+	YES
28	miR-183-5p	+	+	+	+	YES
29	miR-185-5p	+	+	+	+	YES
30	miR-192-5p	+	+	+	+	NO
31	miR-193a-3p	+	+	+	+	NO
32	miR-193a-5p	+	+	+	+	YES
33	miR-193b-5p	+	+	+	NO	NO
34	miR-194-5p	+	+	+	+	YES
35	miR-210-3p	+	+	+	+	NO
36	miR-218-2-3p	+	+	NO	NO	YES***
37	miR-320b	+	+	+	NO	YES***
38	miR-324-5p	+	+	+	+	YES
39	miR-328-3p	+	+	+	+	NO
40	miR-331-5p	+	NO	n/a	n/a	NO
41	miR-339-5p	+	+	+	+	YES
42	miR-340-5p	+	+	+	+	YES
43	miR-362-5p	+	+	NO	NO	NO
44	miR-378a-5p	+	+	NO	NO	NO
45	miR-424-3p	+	+	NO	NO	NO
46	miR-454-5p	+	NO	n/a	n/a	NO
47	miR-486-3p	+	+	+	+	YES
48	miR-486-5p	+	+	+	+	YES
49	miR-488-3p	+	+	+	+	YES
50	miR-491-5p	+	+	+	+	YES
51	miR-494-3p	+	+	+	+	NO
52	miR-501-5p	+	+	+	+	YES
53	miR-505-5p	+	NO	n/a	n/a	YES***
54	miR-550a-3p	+	+	NO	NO	NO
55	miR-571	+	NO	n/a	n/a	NO
56	miR-573	+	+	NO	NO	NO
57	miR-577	+	NO	n/a	n/a	NO
58	miR-589-3p	+	NO	n/a	n/a	YES***
59	miR-596	+	+	NO	NO	NO
60	miR-598-3p	+	NO	n/a	n/a	NO
61	miR-605-5p	+	+	+	+	YES
62	miR-628-5p	+	+	+	NO	YES
63	miR-629-3p	+	NO	n/a	n/a	NO

#	miRNA	miRBase accession	Strong experimental evidence of miRNA-mRNA interactions *	Interaction with the network **	Node degree in the network ≥ 3	Selected for validation
64	miR-661	+	+	+	+	YES
65	miR-766-3p	+	+	+	NO	YES
66	miR-941	+	+	NO	NO	NO
67	miR-1201	NO	n/a	n/a	n/a	NO
68	miR-1275	+	NO	n/a	n/a	YES***
69	miR-1290	+	+	+	NO	YES
**Total number of miRNAs:**	**69**	**68**	**57**	**47**	**39**	**44**

* Experimentally validated miRNA-target interactions from the MIENTURNET web tool (using miRTarBase website release 7.0 from September 2017).

** Working network created with MIENTURNET web tool.

*** miRNAs selected based on literature review (irrespective of bioinformatic analysis).

Supplementary Table 3 (Table S3)

List of analyzed miRNAs and small RNAs for miRNA validation with Custom TaqMan™ MicroRNA Cards.

#	Assay name	Assay Type	TaqMan™ MicroRNA Assay ID
1	let-7f-5p	target	000382
2	miR-9-5p	target	000583
3	miR-18a-3p	target	002423
4	miR-23a-3p	target	000399
5	miR-25-5p	target	002442
6	miR-26a-5p	target	000405
7	miR-26b-5p	target	000407
8	miR-33a-3p	target	002136
9	miR-34b-3p	target	002102
10	miR-95-3p	target	000433
11	miR-96-5p	target	000186
12	miR-99b-5p	target	000436
13	miR-100-3p	target	002142
14	miR-100-5p	target	000437
15	miR-101-3p	target	002253
16	miR-106b-3p	target	002380
17	miR-125a-3p	target	002199
18	miR-135b-3p	target	002159
19	miR-139-5p	target	002289
20	miR-141-5p	target	002145
21	miR-183-5p	target	002269
22	miR-185-5p	target	002271
23	miR-193a-3p	target	002250
24	miR-193a-5p	target	002366
25	miR-194-5p	target	000493
26	miR-218-2-3p	target	002294
27	miR-320b	target	002844
28	miR-324-5p	target	000539
29	miR-339-5p	target	002257
30	miR-340-5p	target	002258
31	miR-424-3p	target	002309
32	miR-486-3p	target	002093
33	miR-486-5p	target	001278
34	miR-488-3p	target	002357
35	miR-491-5p	target	001630
36	miR-501-5p	target	001047
37	miR-505-5p	target	002087
38	miR-589-3p	target	001543
39	miR-605-5p	target	001568
40	miR-628-5p	target	002433
41	miR-661	target	001606
42	miR-766-3p	target	001986
43	miR-1275	target	002840
44	miR-1290	target	002863
45	miR-30e-3p	endogenous control	000422
46	RNU48	endogenous control	001006
47	U6 snRNA	endogenous control	001973
48	U6 snRNA	endogenous control	001973

Supplementary Table 4 (Table S4)

Average fold change values of significantly differentially expressed miRNAs in HGSOC cell lines with (Custom TaqMan™ MicroRNA Cards). Basal expression in the absence of inhibitors was calculated in untreated PEO1-OR and PEO4 cells relative to untreated PEO1 cells. Expression in response to treatment was calculated in all HGSOC cell lines relative to respective untreated controls. Significantly down- and upregulated miRNAs (absolute fold change ≥ 1.5 and $p < 0.05$) were highlighted with blue and red, respectively. n/d – not detected ($C_T \geq 32$ in untreated control cells)

miRNA	BASAL LEVELS		RESPONSE TO TREATMENT														
			PEO1					PEO1-OR					PEO4				
	PEO1-OR	PEO4	O	A	C	O+A	O+C	O	A	C	O+A	O+C	O	A	C	O+A	O+C
let-7f-5p	-1.31	-1.28	1.40	1.29	1.92	**2.04**	1.21	-1.42	-1.86	-1.63	-1.41	-1.22	-1.08	-1.17	-1.07	-1.53	-1.05
miR-9-5p	**1.59**	**1.62**	1.75	1.10	1.63	1.14	1.09	-1.06	1.03	-1.07	-1.06	-1.20	-1.00	-1.32	-1.34	-1.13	-1.09
miR-23a-3p	1.02	-1.25	1.05	-1.23	1.19	1.11	1.21	-1.24	-1.00	1.11	1.01	1.24	-1.17	-1.31	-1.19	-1.25	-1.24
miR-25-5p	-1.37	**-2.16**	1.22	1.01	2.36	2.08	**2.91**	*n/d*	*n/d*	*n/d*	*n/d*	*n/d*	*n/d*	*n/d*	*n/d*	*n/d*	*n/d*
miR-33a-3p	-1.14	1.52	-1.03	-1.05	1.15	-1.43	-1.18	-1.24	-1.21	-1.01	-1.06	**-1.84**	1.07	**-2.07**	1.00	-1.43	-1.48
miR-34b-3p	-1.04	**-1.70**	**1.61**	1.44	-1.11	1.01	1.04	-1.04	-1.07	-1.02	-1.31	-1.21	-1.01	1.39	-1.07	1.02	-1.07
miR-95-3p	**-6.37**	**-6.14**	1.26	-1.14	-1.65	-1.21	-1.16	1.14	-1.14	1.47	**1.99**	**2.28**	-1.04	-1.36	-1.03	1.14	1.39
miR-96-5p	-1.39	**-1.65**	-1.06	-1.33	-1.12	1.05	1.00	**-2.20**	-1.24	-1.31	-1.25	1.10	*n/d*	*n/d*	*n/d*	*n/d*	*n/d*
miR-99b-5p	**-1.86**	**-2.12**	-1.05	-1.22	-1.51	-1.06	-1.09	1.01	-1.11	-1.20	-1.19	-1.05	1.16	-1.22	-1.27	-1.11	-1.51
miR-100-3p	**-7.58**	**-3.85**	1.15	-1.13	**-1.95**	**-1.83**	**-2.44**	1.10	1.18	-1.88	-1.04	-1.84	-1.02	-1.77	-1.31	-1.09	-1.04
miR-100-5p	**-2.41**	**-2.55**	1.24	-1.04	-1.11	1.06	1.08	1.05	-1.20	-1.21	-1.11	-1.14	1.03	-1.13	-1.18	-1.11	-1.18
miR-101-3p	-1.22	1.28	1.00	-1.38	-1.52	-1.97	**-2.26**	-1.03	1.09	-2.11	-1.11	1.08	-1.20	-1.51	-1.39	-1.08	-1.25
miR-106b-3p	-1.20	-1.54	-1.41	**-1.54**	-1.43	-1.01	-1.45	-1.17	-1.05	-1.42	1.05	-1.29	-1.22	-1.26	-1.64	-1.10	**-1.79**
miR-125a-3p	**-2.52**	-1.98	-1.39	1.03	-1.01	-1.56	**-2.53**	*n/d*	*n/d*	*n/d*	*n/d*	*n/d*	-1.72	-2.01	-1.68	-1.05	-1.63
miR-135b-3p	-1.29	**-1.80**	-1.47	-1.06	-1.76	-1.74	**-3.22**	-1.25	-1.00	-1.30	-1.46	-1.60	-1.02	-1.01	1.03	-1.24	1.08
miR-139-5p	-2.37	**-3.88**	1.16	-1.09	1.19	**2.05**	1.52	*n/d*	*n/d*	*n/d*	*n/d*	*n/d*	*n/d*	*n/d*	*n/d*	*n/d*	*n/d*
miR-183-5p	-1.58	**-2.02**	1.54	1.48	1.54	1.89	**2.25**	-1.05	-1.11	-1.21	1.40	1.07	-1.34	-1.60	-1.55	1.05	-1.10
miR-185-5p	1.25	1.08	1.23	1.00	1.05	1.61	-1.00	-1.14	**-1.54**	-1.10	-1.05	-1.22	1.17	1.11	1.19	1.13	-1.13
miR-193a-3p	**-2.31**	-1.35	1.34	1.53	-1.23	**2.41**	1.14	1.19	1.06	-1.10	-1.03	-1.04	1.31	-1.10	-1.41	2.29	**1.61**
miR-324-5p	-1.02	-1.15	**-2.02**	-1.66	**-2.08**	-1.79	**-2.65**	-1.11	**-1.58**	-1.34	-1.41	**-1.59**	-1.14	-1.50	-1.44	-1.33	-1.31
miR-339-5p	-1.59	**-1.77**	1.11	-1.16	-1.20	-1.29	-2.17	1.14	1.04	1.08	-1.04	-1.38	-1.09	-1.22	1.09	-1.33	**-1.62**
miR-340-5p	-1.17	-1.30	-1.85	-1.71	-1.89	-1.72	**-3.35**	1.03	-1.34	-1.08	-1.06	-1.30	-1.07	-1.23	**-1.58**	-1.15	-1.30
miR-424-3p	**-1.99**	**-2.72**	-1.10	-1.11	-1.04	-1.30	-1.68	-1.89	1.11	1.12	1.33	**-1.54**	-1.23	-1.04	-1.37	-1.16	-1.42
miR-486-3p	-1.26	-7.27	**-2.98**	**-3.68**	**-2.31**	-1.83	-2.13	*n/d*	*n/d*	*n/d*	*n/d*	*n/d*	*n/d*	*n/d*	*n/d*	*n/d*	*n/d*
miR-486-5p	**-10.1**	**-8.83**	-1.51	-1.70	-1.63	**-2.78**	**-7.39**	**-2.84**	-1.41	**-2.57**	**-3.22**	**-10.9**	-1.44	-1.57	-1.25	**-5.47**	**-7.34**
miR-491-5p	-1.54	**-3.19**	1.28	1.33	1.83	1.11	1.22	-1.57	1.01	-1.10	-1.15	1.04	**1.89**	1.13	1.46	**1.99**	1.43
miR-505-5p	**-1.74**	**-1.71**	1.12	-1.90	-2.42	-1.60	-1.57	*n/d*	*n/d*	*n/d*	*n/d*	*n/d*	*n/d*	*n/d*	*n/d*	*n/d*	*n/d*
miR-605-5p	-1.84	-1.39	-1.69	-2.41	-1.23	-1.57	-1.03	1.38	-1.03	1.34	1.15	-1.13	**-1.76**	-1.58	-1.48	-1.32	**-1.84**
miR-628-5p	1.57	1.22	**-2.15**	**-2.44**	**-2.48**	-1.79	-1.55	1.51	-1.08	-1.29	1.02	-1.09	1.01	-1.11	-1.29	1.08	-1.16
miR-766-3p	-1.05	-1.34	1.73	1.16	-1.10	1.25	-1.16	**1.58**	1.30	1.11	1.12	-1.23	1.55	-1.08	1.22	1.34	**-2.13**
miR-1275	-1.56	-1.83	1.65	-1.03	1.21	1.80	1.46	1.43	1.31	1.22	**1.84**	1.56	1.39	1.05	1.16	1.43	1.09
miR-1290	**-3.06**	-1.42	1.08	-1.04	1.18	1.17	1.21	1.30	1.57	**2.48**	**2.20**	**3.37**	-1.26	-1.20	1.42	-1.14	-1.03

Supplementary Table 5 (Table S5)

Top significantly enriched pathways (Reactome) and biological processes (GO:BP) associated with target genes of dysregulated miRNAs in untreated PEO1-OR cells.

	**Cell Line:**	PEO1-OR		
	**Treatment:**	Basal expression (in the absence of inhibitors)		
**#**	**Reactome pathway**	**Adj. *p* value**	**Hit number**	**Target genes**
1	Signaling by FGFR	1.21×10^{-6}	8	*UBC, TNRC6A, TNRC6B, MTOR, CREB1, CDKN1A, CALM1, PTEN*
2	Signaling by EGFR	2.16×10^{-6}	8	*UBC, TNRC6A, TNRC6B, MTOR, CREB1, CDKN1A, CALM1, PTEN*
3	PIP3 activates AKT signaling	6.77×10^{-6}	6	*TNRC6A, TNRC6B, MTOR, CREB1, CDKN1A, PTEN,*
4	PI3K/AKT activation	7.29×10^{-6}	6	*TNRC6A, TNRC6B, MTOR, CREB1, CDKN1A, PTEN*
5	Signaling by PDGF	1.49×10^{-5}	7	*TNRC6A, TNRC6B, MTOR, CREB1, CDKN1A, CALM1, PTEN,*
6	Signaling by SCF-KIT	3.24×10^{-5}	6	*TNRC6A, TNRC6B, MTOR, CREB1, CDKN1A, PTEN*
7	Signaling by Wnt	9.81×10^{-5}	7	*UBC, TNRC6A, TNRC6B, CSNK1A1, CALM1, XPO1, CLTC*
8	beta-catenin independent WNT signaling	1.91×10^{-4}	5	*UBC, TNRC6A, TNRC6B, CALM1, CLTC*
9	Downregulation of TGF-beta receptor signaling	3.50×10^{-4}	3	*UBC, XPO1, PPP1CB*
10	Signaling by TGF-beta Receptor Complex	4.24×10^{-4}	4	*UBC, XPO1, PPP1CB, SKI*
11	Cyclin D associated events in G1	5.02×10^{-4}	3	*UBC, CDKN1A, CDK6*
12	TGF-beta receptor signaling activates SMADs	5.68×10^{-4}	3	*UBC, XPO1, PPP1CB*
13	Mitotic G1-G1/S phases	2.61×10^{-4}	4	*UBC, MCM4, CDKN1A, CDK6*
14	Cell Cycle, Mitotic	6.51×10^{-3}	6	*UBC, MCM4, CDKN1A, XPO1, PPP1CB, CDK6*
15	DNA Replication	8.59×10^{-3}	3	*UBC, MCM4, CDKN1A*
**#**	**GO: Biological process**	**Adj. *p* value**	**Hit number**	**Target genes**
1	EGFR signaling pathway	1.32×10^{-3}	5	*UBC, MTOR, CREB1, PTEN, CLTC*
2	regulation of TGF-beta receptor signaling pathway	2.02×10^{-3}	4	*UBC, XPO1, PPP1CB, SKI*
3	G1/S transition of mitotic cell cycle	2.11×10^{-3}	5	*UBC, MCM4, CDKN1A, PTEN, CDK6*
4	cellular response to stress	3.75×10^{-3}	12	*UBC, TERF2, TNRC6A, MTOR, CREB1, VCP, NDRG1, EGR1, CDKN1A, FEM1B, CDK6, HUWE1,*
5	cell cycle checkpoint	4.35×10^{-3}	5	*UBC, NDRG1, MCM4, CDKN1A, FEM1B*
6	Notch signaling pathway	5.74×10^{-3}	4	*UBC, TNRC6A, TNRC6B, CDK6*
7	negative regulation of cellular metabolic process	8.31×10^{-3}	11	*UBC, TERF2, TNRC6A, MTOR, CREB1, ATXN1, EGR1, CDKN1A, XPO1, PTEN, SKI*
8	DNA damage response, signal transduction by p53 class mediator	8.48×10^{-3}	3	*UBC, NDRG1, CDKN1A*
9	signal transduction in response to DNA damage	9.14×10^{-3}	3	*UBC, NDRG1, CDKN1A*
10	DNA damage checkpoint	9.55×10^{-3}	3	*UBC, CDKN1A, FEM1B*
11	response to DNA damage stimulus	9.55×10^{-3}	7	*UBC, TERF2, VCP, NDRG1, CDKN1A, FEM1B, HUWE1*
12	S phase of mitotic cell cycle	9.55×10^{-3}	3	*UBC, MCM4, CDKN1A,*
13	DNA integrity checkpoint	9.92×10^{-3}	3	*UBC, CDKN1A, FEM1B*
14	negative regulation of cell cycle	1.24×10^{-2}	5	*UBC, MCM4, CDKN1A, PTEN, CDK6*
15	positive regulation of cell proliferation	1.49×10^{-2}	6	*ESR1, MTOR, EGR1, CDKN1A, PTEN, CDK6*

Supplementary Table 6 (Table S6)

Top significantly enriched pathways (Reactome) associated with target genes of dysregulated miRNAs in PEO1-OR cells in response to combinations of olaparib with the ATR/CHK1 pathway inhibitors.

#	Reactome pathway	Adj. *p* value	Hit number	Target genes
	**Cell Line:**	PEO1-OR		
	**Treatment:**	Olaparib combination with ATRi or CHK1i (O + A and O + C)		
1	beta-catenin independent WNT signaling	0.0252	3	*TNRC6A, CALM1, CLTC*
2	Loss of Function of TGFBR1 in Cancer	0.0406	1	*SMAD2*
3	Post-transcriptional silencing by small RNAs	0.0406	1	*TNRC6A*
4	Signaling by TGF-beta Receptor Complex in Cancer	0.0406	1	*SMAD2*
5	Caspase-mediated cleavage of cytoskeletal proteins	0.0406	1	*VIM*
6	Chk1/Chk2(Cds1) mediated inactivation of Cyclin B:Cdk1 complex	0.0406	1	*YWHAQ*
7	G2/M DNA damage checkpoint	0.0406	1	*YWHAQ*
8	MicroRNA (miRNA) biogenesis	0.0406	1	*POLR2A*
9	Downregulation of TGF-beta receptor signaling	0.0406	1	*SMAD2*
10	VEGFR2 mediated cell proliferation	0.0406	1	*CALM1*
11	Transcriptional regulation by small RNAs	0.0406	2	*TNRC6A, POLR2A*
12	Downstream signaling of activated FGFR1	0.0406	2	*TNRC6A, CALM1*
13	Signaling by EGFR	0.0406	2	*TNRC6A, CALM1*
14	Signaling by PDGF	0.0406	2	*TNRC6A, CALM1*
15	NGF signaling via TRKA from the plasma membrane	0.0406	2	*TNRC6A, CALM1*

Supplementary Table 7 (Table S7)

Experimentally validated targets of differentially expressed miRNAs from minimal subnetworks that maximally connect seeds in the PEO1-OR cell line (established with miRNet 2.0).

Basal expression (in the absence of inhibitors)		Olaparib combinations with ATRi or CHK1i (O + A and O + C)	
**miRNA**	**Target genes**	**miRNA**	**Target genes**
**miR-9-5p**	*UBC, TNRC6A, CSNK1A1, ESR1, CREB1, NDRG1, DDX21, ITGA5, EGR1, MCM4, CDKN1A, NRC1, CALM1, XPO1, SRRM2, PPP1CB, VIM, CLTC, VPS4A, HUWE1, HECTD1*	**miR-33a-3p**	*TNRC6A, DDX21, CAB39, CALM1, IRF4*
**miR-95-3p**	*MTOR, CREB1, MCM4, CDKN1A, CALM1, SRRM2, VIM, SKI, ZNF131*	**miR-95-3p**	*POLR2A, CALM1, SRRM2, VIM, SCAF4, ZNF131*
**miR-99b-5p**	*TNRC6B, MTOR, ITGA5, MCM4, XPO1, MGA, PPP1CB, VPS4A*	**miR-324-5p**	*TNRC6A, SMAD2, POLR2A, YWHAQ*
**miR-100-3p**	*FN1, PNRC1, SMARCC1, PPP1CB, HNRNPH1, SKI, HECTD1*	**miR-424-3p**	*TNRC6A, VIM, UBE2Z, CLTC*
**miR-100-5p**	*CDKN1A, EEF1D, PNRC1, MGA, PPP1CB, FEM1B, CDK6*	**miR-486-5p**	*DDX21, SMAD2, SRRM2, VIM, PUM2*
**miR-125a-3p**	*UBC, TERF2, FN1, EGR1, MCM4, EEF1D, SRRM2, PPP1CB, CDK6, ARPC2, HUWE1, SKI*	**miR-1275**	*SMAD2, IRF4, UBE2Z, SCAF4, RPP25*
**miR-193a-3p**	*FN1, TNRC6B, ESR1, ATXN1, ARF6, CALM1, PTEN, CLTC, CDK6, HECTD1*	**miR-1290**	*CAB39, YWHAQ, PUM2, CLTC, RPP25, ZNF131*
**miR-424-3p**	*TNRC6A, CSNK1A1, MGA, SMARCC1, VIM, PTEN, CLTC, CDK6, ARPC2, HUWE1*		
**miR-486-5p**	*TERF2, DDX21, ARF6, XPO1, SRRM2, VIM, PTEN, FEM1B, HNRNPH1*		
**miR-505-5p**	*TNRC6B, CSNK1A1, EGR1, CDKN1A, EEF1D, SRRM2, HUWE1, SKI*		
**miR-1290**	*VCP, NDRG1, ATXN1, FEM1B, CLTC, VPS4A, ARPC2, HUWE1, ZNF131, HECTD1*		

Supplementary Table 8 (Table S8)

Identification of hub nodes using CytoHubba plug-in based on the minimal miRNA-mRNA networks using maximal clique centrality (MCC) algorithm. The top 10 hub miRNA targets with the highest connectivity were assigned as potential hub genes (underlined). O – olaparib, A – ATRi, C – CHK1i.

PEO1-OR (untreated)			PEO1-OR (O + A and O + C)			PEO1 (O + A and O + C)		
**Rank**	**Node name**	**Score**	**Rank**	**Node name**	**Score**	**Rank**	**Node name**	**Score**
**1**	UBC	274	**1**	miR-486-5p	6	**1**	UBC	229
**2**	miR-9-5p	138	**1**	SRRM2	6	**2**	FN1	222
**3**	ESR1	126	**1**	miR-1290	6	**3**	ELAVL1	204
**4**	SRRM2	94	**1**	VIM	6	**4**	YWHAG	144
**5**	VCP	80	**1**	miR-95-3p	6	**5**	SRSF1	129
**6**	DDX21	64	**6**	miR-33a-3p	5	**6**	CAND1	96
**7**	VIM	63	**6**	YWHAQ	5	**7**	hsa-let-7f-5p	80
**8**	CALM1	51	**6**	miR-1275	5	**8**	HNRNPH1	51
**9**	FN1	46	**9**	miR-324-5p	4	**9**	miR-125a-3p	50
**10**	CDKN1A	42	**9**	CALM1	4	**10**	MYC	36
**11**	HUWE1	36	**9**	miR-424-3p	4	**11**	CDKN1B	26
**12**	miR-125a-3p	32	**9**	DDX21	4	**12**	ACTN4	22
**13**	EEF1D	27	**13**	SMAD2	3	**13**	SMAD2	21
**14**	TERF2	24	**13**	TNRC6A	3	**14**	ADNP	18
**15**	CLTC	21	**13**	CLTC	3	**14**	miR-101-3p	18
**16**	miR-100-5p	18	**16**	SCAF4	2	**14**	miR-324-5p	18
**17**	EGR1	17	**16**	POLR2A	2	**17**	miR-139-5p	16
**18**	CREB1	16	**16**	RPP25	2	**17**	miR-183-5p	16
**18**	NDRG1	16	**16**	CAB39	2	**17**	CRK	16
**20**	CDK6	15	**16**	ZNF131	2	**20**	SEC24C	15
**20**	ARF6	15	**16**	IRF4	2	**21**	RHOA	13
**22**	SMARCC1	14	**16**	PUM2	2	**22**	miR-340-5p	10
**22**	miR-193a-3p	14	**16**	UBE2Z	2	**23**	PIK3R1	9
**22**	miR-486-5p	14			6	**23**	VIM	9
**25**	miR-1290	13			6	**23**	TERF2	9
**26**	miR-95-3p	11				**23**	TNRC6B	9
**27**	XPO1	10				**23**	NOTCH1	9
**27**	HNRNPH1	10				**23**	PLCG1	9
**27**	miR-424-3p	10				**29**	miR-193a-3p	8
**30**	miR-99b-5p	8				**30**	miR-135b-3p	7
**30**	miR-505-5p	8				**30**	miR-486-5p	7
**32**	PPP1CB	7				**32**	RBM28	6
**32**	PTEN	7				**32**	TNRC6A	6
**32**	ARPC2	7				**32**	miR-25-5p	6
**32**	SKI	7				**32**	miR-100-3p	6
**32**	miR-100-3p	7				**32**	NF2	6
**32**	ITGA5	7				**37**	FBXW7	5
**38**	MTOR	6				**37**	ZNF652	5
**38**	MCM4	6				**37**	ATXN1L	5
**40**	HECTD1	5				**40**	UBE2Z	4
**40**	TNRC6B	5				**40**	NHLRC2	4
**40**	ATXN1	5				**40**	ITSN2	4
**43**	MGA	4				**43**	VPS37A	3
**43**	FEM1B	4						
**43**	VPS4A	4						
**43**	CSNK1A1	4						
**43**	PNRC1	4						
**48**	ZNF131	3						
**48**	TNRC6A	3						

Supplementary Table 9 (Table S9)

Results of stage-wise differential miRNA and gene expression analysis in serous OC patients using filtered data from TCGA-OV dataset (serous OC patients with stage II, III, or IV after pharmaceutical therapy). Gene and miRNA levels were expressed as counts per million (CPM). Statistical significance for non-normally distributed data was evaluated with the Kruskal-Wallis test comparing the medians of three groups followed by Dunn's multiple comparison test (if applicable).

**miRNA**	**Average expression (CPM)**			**Median expression (CPM)**			**Kruskal-Wallis test *p* value**
	**Stage II**	**Stage III**	**Stage IV**	**Stage II**	**Stage III**	**Stage IV**	
**miR-9-5p**	41.6	38.7	27.5	57.2	59.1	33.8	0.2384
**miR-99b-5p**	226675	262851	242556	240102	265850	231672	0.4013
**miR-100-5p**	1848	2982	2238	1932	2395	2141	0.2324
**miR-125a-3p**	39.8	43.2	49.1	36.3	37.7	41.4	0.4617
**miR-324-5p**	47.4	43.6	45.6	44.4	38.9	39.6	0.9585
**miR-424-3p**	11.9	14.6	14.2	9.8	12.2	12.1	0.5566
**miR-486-5p**	492.9	512.6	346.2	328.1	372.9	231.1	0.3533
**miR-505-5p**	35.4	23.2	23.1	34.5	21.3	20.6	0.2263
**Gene**	**Average expression (CPM)**			**Median expression (CPM)**			**Kruskal-Wallis test *p* value**
	**Stage II**	**Stage III**	**Stage IV**	**Stage II**	**Stage III**	**Stage IV**	
HUWE1	385.5	379.6	371.3	358.1	352.8	397.5	0.9030
TNRC6B	74.7	56.2	54.2	70.6	55.6	50.2	0.0647
EEF1D	129.7	112.8	126.6	121.3	109.7	113.7	0.6075
CDK6	28.0	24.2	40.9	16.0	19.6	33.6	0.0255
CSNK1A1	86.0	79.1	84.4	89.4	75.7	83.9	0.3994
EGR1	297.0	276.4	177.9	272.0	211.0	148.9	0.2884
CDKN1A	54.6	51.8	41.7	44.9	45.17	39.8	0.4951
ITGA5	47.02	61.1	49.24	35.5	47.5	41.8	0.4789
SRRM2	973.8	754.7	756.4	933.1	745.0	754.6	0.0975
PTEN	89.1	101.2	94.6	88.0	96.7	103.0	0.6343
SMARCC1	220.0	177.5	175.5	208.6	170.9	185.3	0.1008
MGA	54.5	48.1	49.3	52.0	47.0	51.1	0.3749
VIM	239.8	418.0	390.1	250.2	360.9	361.8	0.0271
TNRC6A	76.7	58.8	58.93	67.2	55.9	53.1	0.0373
YWHAQ	400.7	438.7	421.2	395.2	407.4	352.2	0.6053
CLTC	298.1	285.6	299.9	295.3	271.2	260.3	0.8709
UBE2Z	158.6	157.4	150.3	156.9	150.3	137.7	0.7519
SMAD2	158.6	157.4	150.3	156.9	150.3	137.7	0.7519
POLR2A	271.0	220.4	195.1	270.2	207.3	186.0	0.0074

Supplementary Table 10 (Table S10)

Validation of endogenous control genes stable expression in HGSOC cell lines for RT-qPCR data normalization from Custom TaqMan™ MicroRNA Cards. Average C_T values are means for all tested treatments (control, O, A, C, O+A, and O+C) among cell lines. Significant differences in average C_T values between treatments for each gene and cell line were evaluated with ordinary one-way ANOVA. O – olaparib, A – ATRi, C – CHK1i.

Endogenous Control	Cell Line	Average C_T	SD of C_T	Significant Difference Among Treatments	C_T MIN	C_T MAX	C_T Range
**U6 snRNA**	PEO1	20.10	0.14	NO (p = 0.6844)	19.91	20.29	0.38
	PEO4	20.00	0.22	NO (p = 0.6553)	19.52	20.16	0.64
	PEO1-OR	20.18	0.07	NO (p = 0.9734)	20.06	20.26	0.20
**RNU48**	PEO1	20.34	0.08	NO (p = 0.9480)	20.24	20.47	0.24
	PEO4	20.09	0.10	NO (p = 0.8417)	20.00	20.29	0.29
	PEO1-OR	20.08	0.08	NO (p = 0.6809)	19.98	20.17	0.19
**miR-30e-3p**	PEO1	27.73	0.22	NO (p = 0.2772)	27.34	28.01	0.67
	PEO4	27.46	0.17	NO (p = 0.6857)	27.18	27.67	0.50
	PEO1-OR	27.34	0.12	NO (p = 0.9296)	27.12	27.51	0.39
